# Transport variability over the Hawkesbury Shelf (31.5–34.5°S) driven by the East Australian Current

**DOI:** 10.1371/journal.pone.0241622

**Published:** 2020-11-05

**Authors:** Nina Ribbat, Moninya Roughan, Brian Powell, Shivanesh Rao, Colette Gabrielle Kerry

**Affiliations:** 1 Coastal and Regional Oceanography Lab, School of Mathematic and Statistics, UNSW Sydney, Sydney, NSW, Australia; 2 Department of Oceanography, University of Hawaii, Honolulu, HI, United States of America; Universidade de Aveiro, PORTUGAL

## Abstract

The Hawkesbury Bioregion located off southeastern Australia (31.5–34.5^o^S) is a region of highly variable circulation. The region spans the typical separation point of the East Australian Current (EAC), the western boundary current that dominates the flow along the coast of SE Australia. It lies adjacent to a known ocean warming hotspot in the Tasman Sea, and is a region of high productivity. However, we have limited understanding of the circulation, temperature regimes and shelf transport in this region, and the drivers of variability. We configure a high resolution (750m) numerical model for the Hawkesbury Shelf region nested inside 2 data assimilating models of decreasing resolution, to obtain the best estimate of the shelf circulation and transport over a 2-yr period (2012–2013). Here we show that the transport is driven by the mesoscale EAC circulation that strengthens in summer and is related to the separation of the EAC jet from the coast. Transport estimates show strong offshore export is a maximum between 32-33^o^S. Median offshore transports range 2.5–8.4Sv seasonally and are a maximum during in summer driven by the separation of the EAC jet from the coast. The transport is more variable downstream of the EAC separation, driven by the EAC eddy field. Onshore transport occurs more frequently off Sydney 33.5–34.5^o^S; seasonal medians range -1.7 to 2.3Sv, with an onshore maximum in winter. The region is biologically productive, and it is a known white shark nursery area despite the dominance of the oligotrophic western boundary current. Hence an understanding of the drivers of circulation and cross-shelf exchange is important.

## 1. Introduction

Along southeastern Australia, the circulation is largely dominated by the poleward flowing East Australian Current (EAC) and its eddy field. Typically, the EAC separates from the coast at ~31.5°S varying between 30.7–32.4^o^S, with maximum excursions between 28–38^o^S [1]. Anticyclonic (warm core eddies) shed from the current every ~90–120 days [1–3] associated with the separation of the jet. The EAC jet is known to strengthen in summer [4], with an associated increase in eddy kinetic energy downstream of the separation point [5].

Upstream of the EAC separation, the circulation is largely anisotropic, dominated by the poleward flowing jet. The shelf circulation in this region has been well studied using surface velocities from HF radar [4, 6] and data from a long-term mooring array [7–9] centred at 30^o^S where the shelf is narrow (~30 km). Cross-shelf exchange in the region is driven by onshore encroachment of the EAC jet [7–11], intermittent wind driven upwelling and downwelling [7–9], and the generation and propagation of frontal eddies [12–14].

Downstream of the EAC separation point, the flow on the shelf is dominated by the dynamic eddy field of the EAC system [15, 16]. Seasonality contributes to only 6% to the overall velocity variability observed at a shelf mooring array off Sydney (~34^o^S). Here, flow variability generally occurs on the mesoscale eddy shedding timescales of 90–120 days [3].

Investigations into the drivers of coastal upwelling along southeastern Australia showed that wind forcing is sporadic, weak (mean 0.09 Nm^-2^) and tends to be downwelling favourable. For example off Sydney (34^o^S) wind forcing was shown to be downwelling favourable 31% of the time, versus 4% upwelling favourable over a 10-year period [17].

Immediately downstream of the separation point, along the Hawkesbury Shelf (31.5-34^o^S) there are few fixed observations, and an understanding of the shelf circulation and the impact of the EAC jet on this circulation is poor. However, this region (including the Stockton Bight) is a known region of high productivity [18], and is recognised as a white shark nursery are [19], hence an understanding of the circulation in the region is important.

To date the circulation in the EAC System has been explored broadly with the use of coarse resolution hydrodynamic models such as the (10 km resolution) BlueLink reanalysis [20, 21], OFES [1] and a higher resolution (2.5–5 km resolution) 22-yr free running ROMS simulation of the EAC System [5]. To improve estimates of the EAC system Kerry et al. [22] developed a 2-yr nested reanalysis that assimilates all available observations in the region [22–24]. This is the most accurate representation of the circulation in the region for the 2012–2013 period. These studies have explored the dynamics of the EAC system, its seasonality and its separation from the coast at the broadscale, however this is the first high resolution modelling study of shelf circulation in the Hawkesbury Shelf Bioregion.

Here we explore the circulation on the continental shelf immediately downstream of the EAC separation point, in order to understand its magnitude, variability and drivers. We quantify the along and cross-shelf transport and its variability over the shelf and explore the seasonal variability to obtain a complete picture of the 3-dimensional circulation and transport along and across the Hawkesbury shelf. To achieve this, we configure a high resolution (750m) hydrodynamic model for the Hawkesbury Shelf (the Hawkesbury Shelf Model, HSM) immediately downstream of the EAC separation point. The specific aims of this work are to:

1. Characterize the mean and variability of the temperature and circulation on the Hawkesbury Shelf over 2012–2013,
2. Evaluate the transport and its variability along and across the shelf, and
3. Determine the regions and drivers of cross-shelf exchange.

We nest the HSM inside the 2-yr reanalysis [22] to ensure the shelf circulation is as accurate as possible. The model configuration is described and evaluated in Section 2, with additional validation provided in the Supporting Documentation. In Section 3 the model is then used to explore the dominant circulation patterns and to quantify the along and cross-shelf transport over the Hawkesbury shelf, and the associated export regions. Context and discussion of drivers are provided in Section 4, followed by limitations and recommendations for future work.

## 2. Materials and methods

### 2.1 Study site

**Fig 1:**
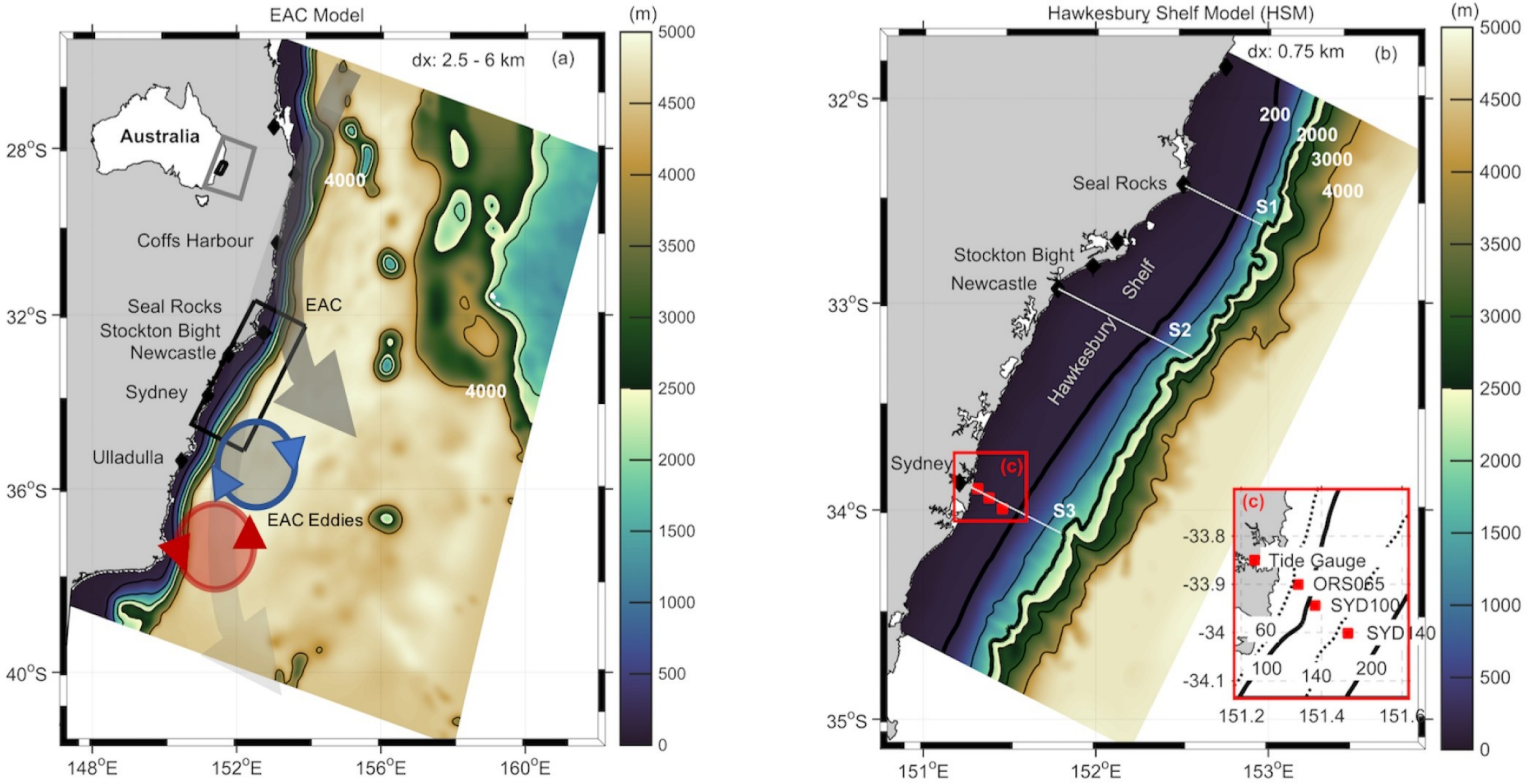
Spatial location, domains and bathymetry of the model grids. (a) East Australian Current (EAC) model domain with schematic of the main EAC flow overlaid, (b) Hawkesbury Shelf Model (HSM) domain. (c) The location of the observations used for model evaluation including the 3 moorings ORS065, SYD100 and SYD140, and the tide gauge station in Sydney Harbour indicated by the red squares. The 3 shore-normal sections from 0–2000 m used in the analysis are shown at Seal Rocks, Newcastle and Sydney, labelled as S1, S2 and S3 respectively. The horizontal cross-shelf resolution of each model is shown as dx. Colour shading in (a) and (b) represents the depth of the bathymetry (m).

The study site is located on the southeast coast of Australia (Fig 1A) with a focus on the Hawkesbury Shelf Region (Fig 1B) that lies immediately downstream of the typical EAC separation zone. Our focus extends from 31.5^o^S to 34.5^o^S from the coast to the 4000m isobath (~ 150 km offshore) at the bottom of the continental slope. In this region the shelf is near its widest, ranging 50–80 km. We focus our analysis on three cross-shelf sections, S1, S2, and S3 located off Seal Rocks (~32.4^o^S), Newcastle (~33^o^S) and Sydney (~34^o^S) respectively. In addition, we investigate exchange between the inner (<100 m), middle (<200 m), and outer shelf (2000 m).

Across the shelf at the Sydney section, (S3) there are 3 long-term oceanographic moorings (Fig 1C). The first, ORS065 is maintained by Sydney Water Corporation since the early 1990s, the other two moorings, SYD100 and SYD140, are deployed and maintained by the NSW node of Australian’s integrated Marine Observing System (NSW-IMOS) since 2008. These moorings deployed in 65m, 100m and 140m respectively provide temperature and velocity information at 2-8m intervals through the water column. Temperature loggers were moored at 4m intervals at the ORS065 and 8m intervals at SYD100 and SYD140. Bottom mounted acoustic Doppler current meters (ADCPs) provided velocity at 2, 4, or 8m resolution at the 3 moorings respectively. All data were recorded at 5 min intervals but were averaged to hourly in this study. Mooring data for model validation at SYD140 and ORS065 are available from Jan 2012-Dec 2013, and at SYD100 from Sept 2012-Dec 2013. The mooring data used for evaluation are described more fully in S1 Table and further information on the array including details regarding instrumentation, sensors, data quality control and processing can be found in [25, 26].

### 2.2 The hydrodynamic model configuration

The modelling framework consists of the high-resolution Hawkesbury Shelf Model (HSM), (Fig 1B), nested within a lower-resolution, eddy-resolving data-assimilating reanalysis of the EAC region configured over a 2-yr period, 2012–2013. Both models are configurations of the Regional Ocean Modelling System (ROMS, V3.4, www.myroms.org). The parent model, (hereafter referred to as the EAC model, Fig 1A) has a variable horizontal grid resolution with 2.5–6 km in the cross-shelf direction (increasing to 6 km offshore of the shelf break) and 5 km resolution in the along-shelf direction for a total of 270 x 315 grid cells and 30 vertical s-layers. The parent model is nested inside BRAN (the BlueLink Reanalysis), a 10 km near global eddy-permitting reanalysis, described in [20, 27]. Full details of the high resolution EAC reanalysis and validation can be found in Kerry et al. [22]. The HSM is one-way nested inside the EAC reanalysis using the method of Mason et al. [28] and is configured over the same 2-yr period (2012–2013). The HSM has a horizontal resolution of 750m for a total of 197 x 477 grid cells. The model’s 30 s-layers are distributed with a higher resolution in the upper ocean to resolve the wind-driven circulation and near the bottom for improved resolution of the bottom boundary layer. The depths of the surface layer in the three different model grids are: BRAN—constant z-level, d = 2.5m, EAC model, d = 0.3–3.5m and HSM d = 0.03–1.67m. S1 Fig shows the improvement associated with the increase in resolution between the three models, including the improvement in resolution of SST (S1A–S1C Fig), grid spacing (S1D–S1F Fig), vertical resolution including improvements in bathymetry and resolution by the coast (S1G–S1I Fig).

Bathymetry was obtained from Geosciences Australia (50 m Multibeam Dataset for Australia [29]). The HSM model was configured carefully to ensure a realistic representation of the shelf and slope topography. Minimum water depth across the domain was limited to 4m for numerical stability with a maximum depth of 4910m. The smoothing method of Sikiric et al. [30] was applied to the bathymetry of the HSM to reduce horizontal pressure gradient errors.

The ROMS HSM was configured with fourth-order centered vertical advection of tracers, fourth-ordered centered vertical advection of momentum, third-order upstream horizontal advection of 3D momentum and third-order upstream horizontal advection of tracers for the mixing of momentum [31]. ROMS uses a split-explicit time stepping scheme [32] and, in this configuration, the barotropic and baroclinic timesteps are 2.1 s and 32 s respectively. The turbulent mixing scheme for the HSM was the Mellor and Yamada scheme 2.5 (MY2.5, [33]). A quadratic drag formulation is used with a constant drag coefficient of 3.0 x 10^−3^ [34]. Instantaneous model outputs are saved in 2-hrly intervals in order to resolve tidal induced motions and daily averages are also saved.

#### 2.2.1 Nesting

To avoid depth mismatches between the parent (EAC model) and child (HSM model) grids, the bathymetries are gradually merged over the regional baroclinic Rossby Radius of 19 km (25 grid cells) along the eastern, northern and southern boundary [28]. The downscaling ratio was between 3:1 and 5:1, varying due to the varying cross-shelf resolution of the EAC model (after Penven et al. [35]). An example of the improvement in resolution achieved is shown along the southern boundary in S2 Fig.

#### 2.2.2 Boundary and initial conditions

Sea surface height, temperature, salinity and current velocity outputs from the parent (EAC) model were obtained every 4 hours from the reanalysis and used as the open boundary for the HSM. The EAC reanalysis also provided the initial conditions for the HSM model. Along the open boundaries the Flather [36] and Chapman [37] conditions were applied to 2D velocities and sea surface height with free-slip boundary conditions on the landward side. For the 3D variables, the radiative boundary condition was used [38] with nudging to the parent grid at time scales of one day.

#### 2.2.3 Forcing

The EAC model and the HSM were forced with the same atmospheric forcing data with a 12 km spatial and 6-hourly temporal resolution. Data was made available by the Australian Community Climate and Earth-System Simulation (ACCESS [39]). By applying the standard bulk flux algorithm [40], ACCESS wind fields, air temperature, relative humidity and air pressure are used to calculate the air-sea fluxes of momentum and buoyancy. Typically, along the southeast coast of Australia, rainfall is low and river inflow has minimal impact on shelf flows except during time of extreme rainfall [41], thus river inflow is neglected. Tidal surface elevation and momentum comprised of the main constituents of the diurnal and semidiurnal tidal band (M2, S2, N2, K2, K1, O1, P1, Q1), derived from the TPXO8 Atlas [42] were applied along the lateral open boundaries of the HSM.

### 2.3 Model evaluation

The parent EAC model has been rigorously validated against observations as presented in Kerry et al. [22]. This reanalysis was shown to successfully capture the mesoscale variability of the EAC System during 2012–2013. We provide some comparison of the parent model with the HSM. Some observations from within the HSM domain (e.g SST and SYD140 mooring data) were assimilated into the EAC model [22]. Assessment of the nested HSM model’s performance was undertaken against observations that were both assimilated and non-assimilated (e.g ORS065 and SYD100) into the parent model. Common statistical measures including the mean, standard deviations (SD) and Root Mean Square Difference (RMSD) and correlations of the temperature and velocity fields (u,v) were assessed against observations to statistically quantify the performance of the HSM. All the statistical analyses were carried out in observation time and space.

#### 2.3.1 Sea Surface Temperature (SST)

SST data from the surface layer of both the EAC model and HSM were compared to SST observations from AVHRR (Advanced-very high-resolution radiometer) L3S daily merged maps from 2012–2013 (http://www.ghrsst.org) at 2 x 2 km resolution. Analysis of local winds reveal that wind driven mixing was likely to have occurred 97% of the time over the period (not shown), hence the use of AVHRR day/night composite as representative of the surface layer of the ocean is justified in this case. Model outputs were re-gridded into observational space and statistical parameters were computed for periods with more than 15% spatial coverage.

**Fig 2:**
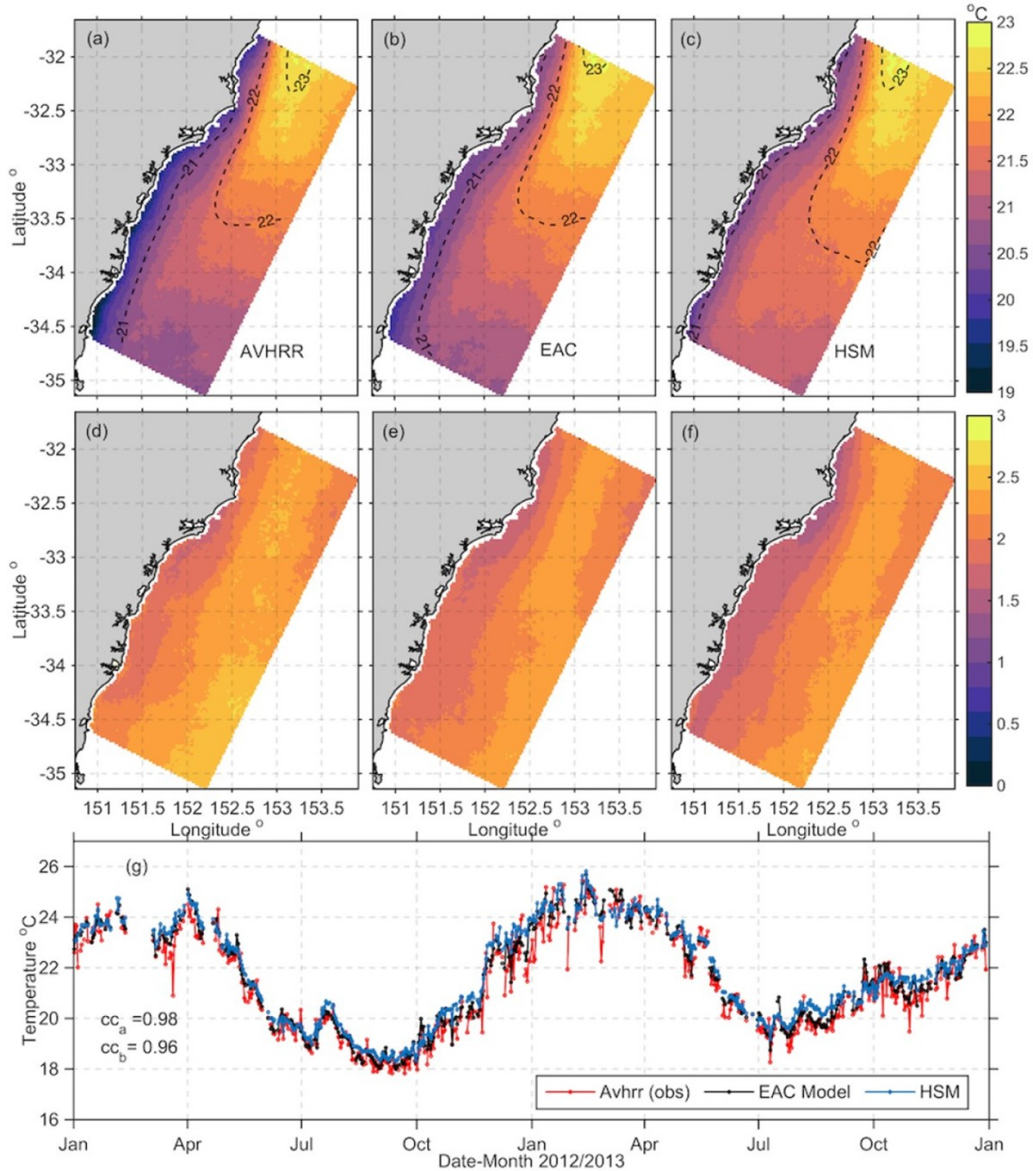
Domain wide 2-yr mean sea surface temperatures (SST) in ^o^C (top row) and their associated standard deviations (middle row) from (a) AVHRR L3S satellite data, (b) EAC model, (c) HSM. Associated standard deviations are shown in (d-f). (g) Time series of spatially averaged SST (^o^C) across the domain for time periods with 15% or more satellite (AVHRR) data over the 2 years. Correlation coefficients are shown where cc_a_ is the correlation between the satellite and EAC model SST and cc_b_ is the correlation between the satellite and HSM SST.

Domain wide 2-yr mean sea surface temperatures (SST) and their associated standard deviations are shown for the EAC and HSM models, as well as the AVHRR data Fig 2. The HSM reproduces the overall pattern of observed SST fields (Fig 2A and 2C) and their variability (Fig 2D and 2F). The parent EAC model performed slightly better, as it assimilates SST data at the surface (Fig 2B and 2E). The correlation between the HSM and the domain average AVHRR SST is very high (0.96), only slightly lower than that of the EAC assimilating model (0.98) with the seasonal cycle well represented over the period. The HSM overestimates the SST slightly, with higher temperatures ranging from 0.5°C in the northeastern part of the domain to 1.25°C in the south, however the spatial temperature patterns are well replicated. The assimilating model reduces the heat fluxes in the analysis, and hence in the free running HSM, SST is slightly higher, despite using the same forcing.

#### 2.3.2 Mesoscale circulation variability

**Fig 3:**
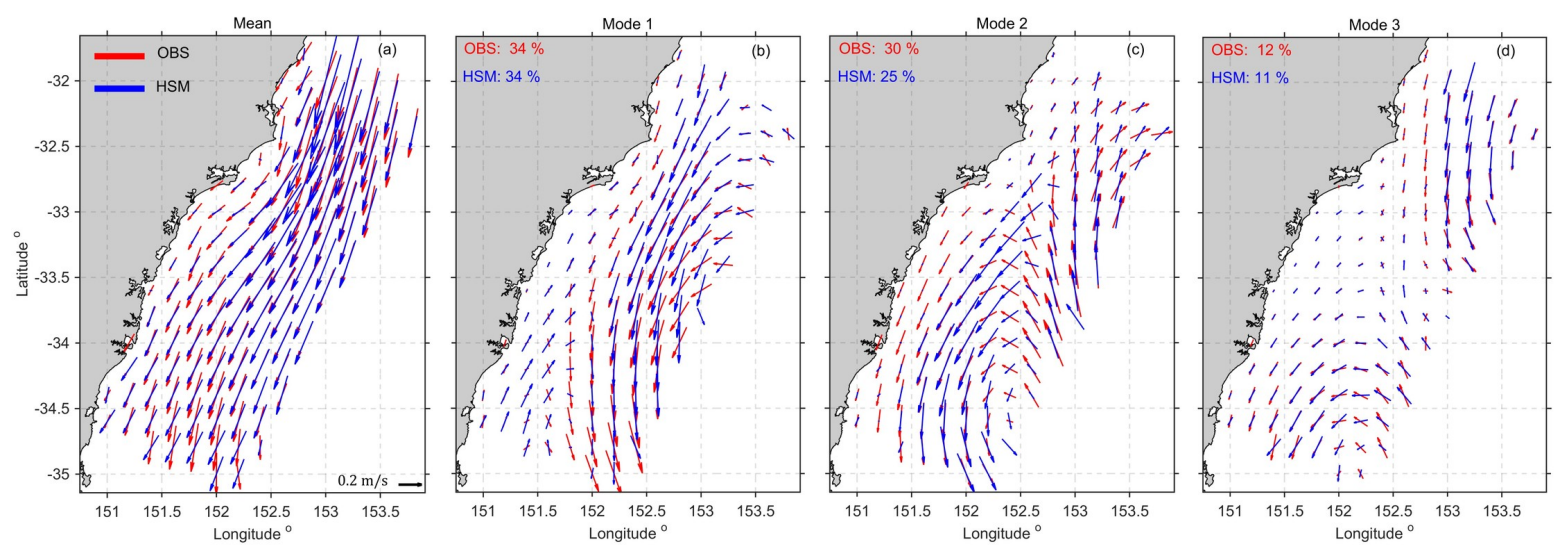
**EOF analysis of daily geostrophic velocities from the HSM (blue) and satellite (red) for 2012–2013.** Spatial structure of the (a) Mean, (b) Mode 1, (c) Mode 2 and (d) Mode 3 showing velocity fields (arrows). Percentages show the variances accounted for by each mode in the model and observations.

To show that the model depicts the mesoscale variability driven by the EAC and its eddy field, geostrophic velocities calculated from the HSM elevations were compared with satellite derived geostrophic velocities [43]. Modelled geostrophic velocities were interpolated onto the altimetry grid with a resolution of 0.25 x 0.25^o^. Empirical Orthogonal Functions (EOFs) were calculated using daily-averaged geostrophic velocity fields for the simulation period (Fig 3). The variance explained by the EOFs agreed well ranging 34%-11% for mode 1–3 (Fig 3). High correlations were obtained between the modelled and observed temporal expansion function of the geostrophic velocity EOFs with correlation coefficients of 0.69 for mode 1, 0.66 for mode 2, and 0.74 for mode 3, (S3 Fig). It is useful to bear in mind that the calculation of geostrophic velocities from altimetric data assumes isotropy, which is not always valid in this region. However, these results show the HSM’s ability to capture the variability driven by the mesoscale circulation.

#### 2.3.3 Barotropic tides

To validate coastal sea level variations simulated by the HSM, modelled tidal phases and amplitudes were compared to observations from the Fort Denison Tide Gauge within Sydney Harbour (Fig 1C), located 1.2 km inside the Estuary in 8.45 m water depth. Within the model grid domain, it was located 4 grid cells (3 km) upstream from the first wet grid cell in the model at 9.7 m water depth resulting in an ~ 10-minute tidal lag. Tidal harmonics from both the modelled and observed sea level elevations were estimated through harmonic analysis [44]. In addition, to quantitatively compare both phases and amplitudes of the data, the absolute RMS error [45] was used (S2 Table). The good agreement between modelled and observed sea level heights (correlation coefficient of 0.93, 95% confidence, S4 Fig) indicate the HSM’s skill in adequately reproducing sea level height variations induced by barotropic tides. The absolute RMS is less that 10% for all constituents, except for K1 and S2 (See S2 Table for further details).

#### 2.3.4 Velocity over the shelf

Current velocities were obtained from acoustic Doppler current profilers (ADCPs) deployed at three moorings as described above and in S1 Table. Both the observed hourly time series and the 2-hrly model output velocities were smoothed with a 38-hour low-pass filter using the PL64 filter [46] to remove high frequency variability and inertial oscillations that may not be captured by the model. The observed and modelled velocities were rotated into along and cross-shelf components.

**Fig 4:**
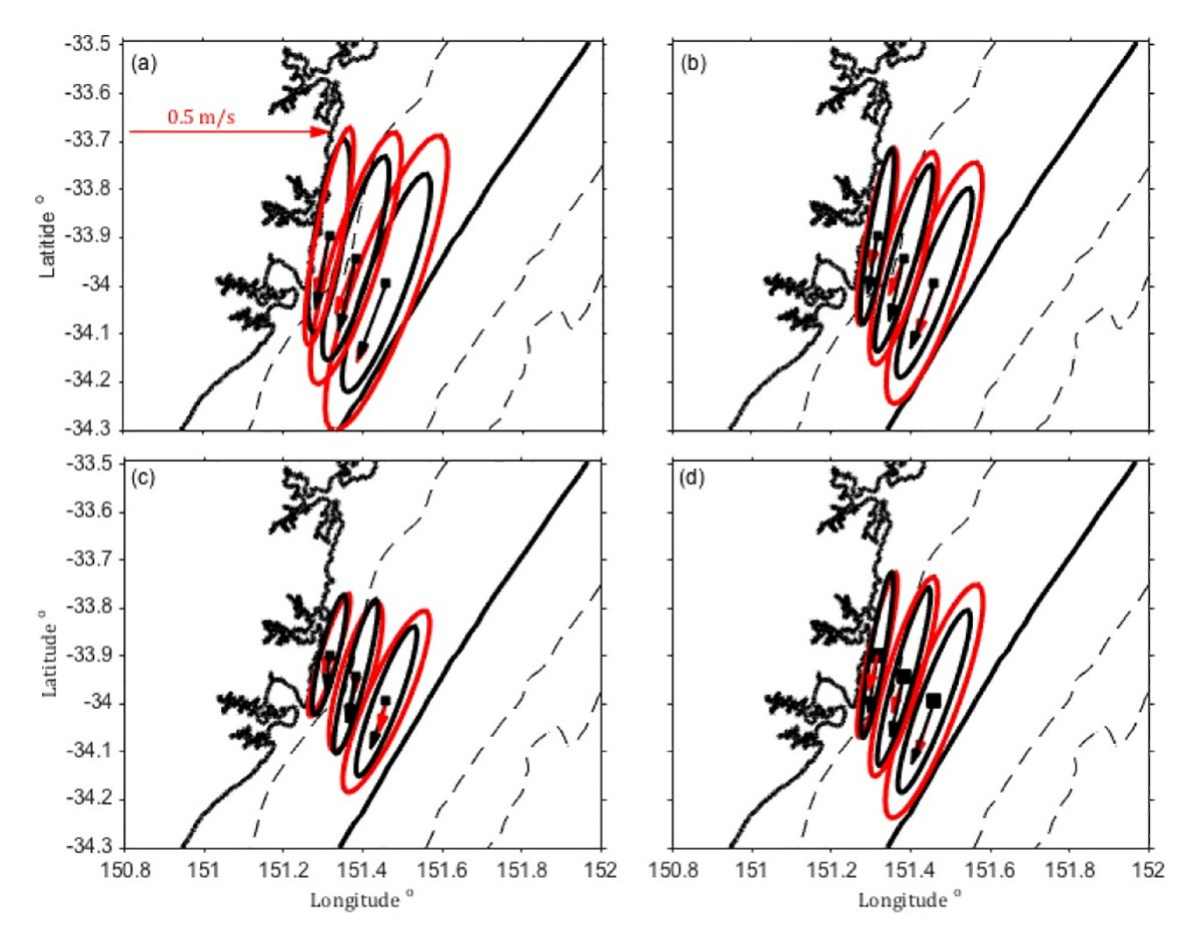
**Mean current vectors and variance ellipses of modelled (HSM, black) and observed (red) velocities for 2012–2013 at the location of the 3 moorings off Sydney.** Dashed lines show the 100, 1000 and 2000 m isobath; the solid black line shows the location of the 200 m isobath. (a) Upper—depth bin, (b) mid—depth bin, (c) bottom—depth bin and (d) full water column, depth averaged velocities in ms^-1^.

Mean velocity and variance ellipses were computed at each mooring location and compared to observations (Fig 4 and S3 Table) to show the HSM’s ability to capture the high-resolution shelf circulation variability. The water column was divided into 3 depth bins of roughly 1/3 of the water column each to represent the upper, mid and lower part of the water column and velocities were depth-averaged for each bin. The HSM performs well through the water column, with good agreement in magnitude, variability and direction at each layer. Results are presented fully in S3 Table. The 2-yr mean surface velocities are weakest inshore, and strongest offshore where the EAC dominates. The flow is broadly anisotropic, representing the poleward EAC flow. The model agrees well with the observations, with the lowest agreement occurring in magnitude of the surface layer at the shelf break, where the velocities are greatest and EAC dominated, here the model underestimates the surface velocities by 0.04 ms^-1^ (S3 Table).

A spectral analysis of the modelled and observed velocities (S5 Fig) shows that the model has similar power at the semidiurnal, diurnal, and inertial frequencies. Although the two year run is not long enough to differentiate between the inertial and diurnal frequencies as the inertial period ranges from 25.4–27.5 hours in the model domain. In addition, super inertial frequencies are not yet resolved well. It has been shown in this region that a 750m resolution model with 6 hourly forcing cannot resolve the fine scale circulation features associated with super inertial frequencies [24].

**Fig 5:**
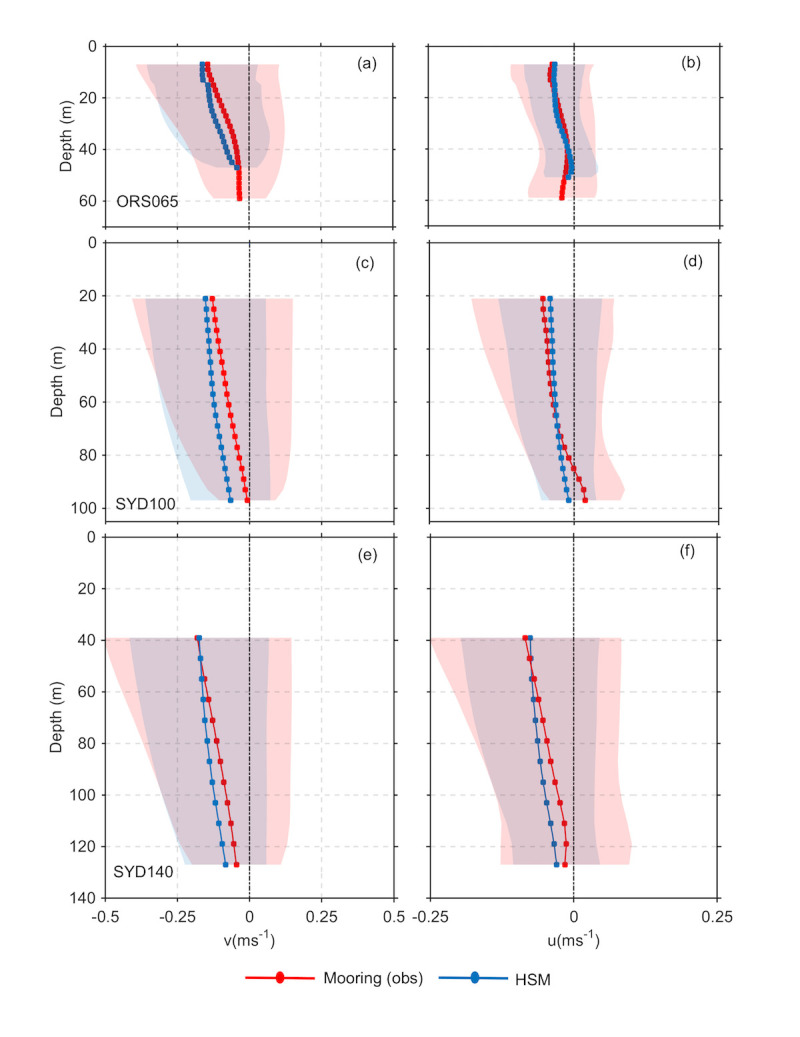
Observed (red) and modelled (black) along shore (v, left) and cross-shore (u, right) velocity profiles (ms^-1^) with depth at the three shelf moorings, ORS065 (a,b), SYD100 (c,d), and SYD140 (e,f). Shading represents the standard deviation and mooring locations are shown in Fig 1.

**Fig 6:**
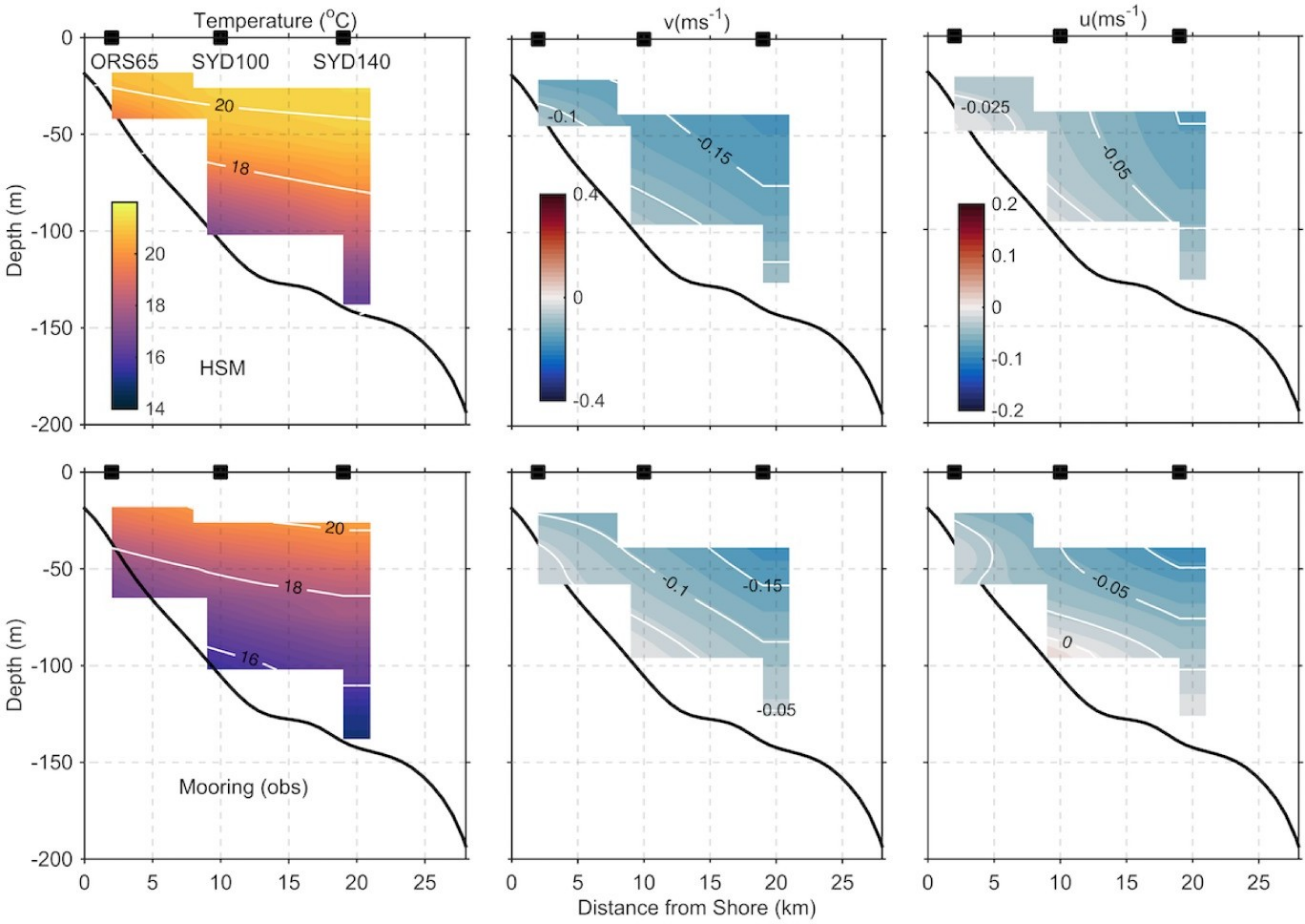
**Modelled (top panel) and observed (bottom panel) cross-sections of the mean temperature (left column), along-shelf velocity (middle column, negative poleward) and cross-shelf velocity (right column, negative onshore) off Sydney (34**^**o**^**S).** The positions of the 3 moorings are indicated by black squares.

Vertical velocity profiles at the mooring locations are shown in Fig 5 for the observed and modelled along and cross-shore velocities. The vertical structure of the modelled velocity fields and the velocity range at the mooring locations matches the observed velocities well, as does the standard deviation. Mean poleward velocities are up to 0.2 ms^-1^ in the surface offshore. Cross shelf velocities are onshore in the mean, at 34^o^S. Cross-sections of 2-yr mean of the observed and modelled along and cross-shore velocities are presented in Fig 6. Showing the structure of the velocity field over the shelf. Mean poleward velocities reach 0.25 ms^-1^ in the surface waters, while cross shelf velocities are slightly onshore on average at this latitude. These results give confidence in the use of the model to understand circulation and transport along and across the shelf.

#### 2.3.5 Temperature over the shelf

Observed temperature data were obtained from the 3 moorings (as described above and in S1 Table). A statistical assessment of the modelled temperature compared with observed sub-surface temperature is presented in a Taylor diagram [47], that simultaneously depicts the correlation coefficients, root mean square differences and normalized standard deviation (S6 Fig). The correlations between the modelled and observed temperatures range 0.4–0.85 and are highest in the upper and lower water column at the mid-shelf moorings. Correlations are lowest mid water column at the offshore site where RMS differences show the model underrepresents the temperature fluctuations (S6 Fig). This is most likely associated with the movement in the thermocline as the EAC moves on and off the shelf. Cross sections of temperature over the shelf are shown in Fig 6 in observation space. The figure shows agreement in the vertical and horizontal structure of temperature across the shelf, and the temperature range (14–21°C) although the model is warmer than the observations by about 1 degree.

## 3. Results

### 3.1 Seasonal velocity variability

**Fig 7:**
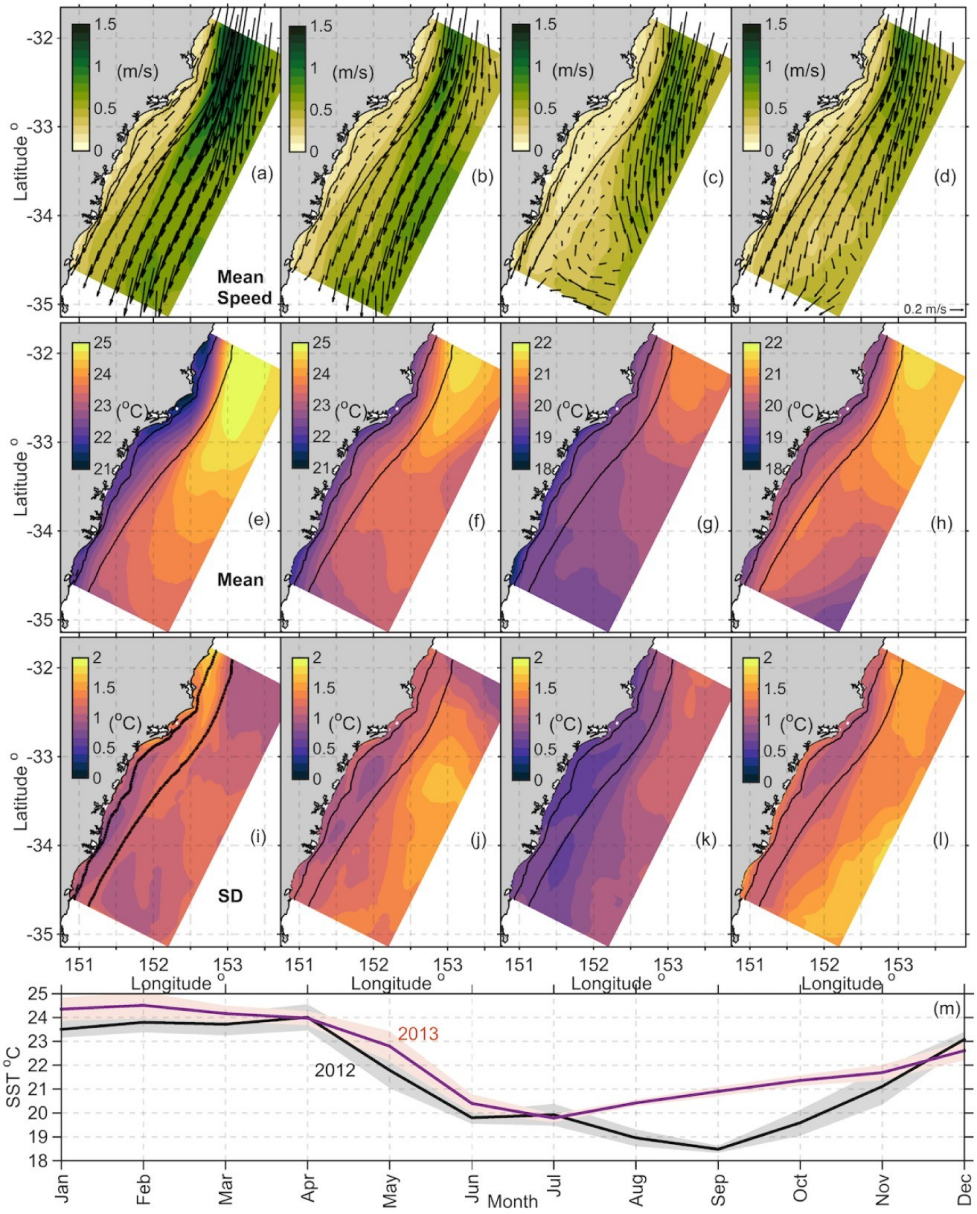
Domain-wide seasonal mean modelled fields. Velocity fields at the surface (a-d). Velocity vectors show the direction and colour shows the magnitude (ms^-1^) of the speed. Every 25^th^ vector is shown for clarity. The reference velocity arrow in the bottom right of each panel represents 0.2 ms^-1^. Domain wide mean SST by season (e-h) and associated standard deviations (i-l) in (^o^C). Solid black lines show the 100 m and 2000 m isobaths. Area averaged SST (m) showing monthly means for 2012 (black), 2013 (red). Shaded areas represent the standard deviations.

Variability in velocity and temperature was investigated seasonally throughout the domain. The surface currents and temperatures were averaged by season for the 2-yr period (2012–2013), (noting that the Austral summer is Dec, Jan and Feb) and are shown in (Fig 7).

Throughout the 2-yr period, the poleward flowing EAC jet dominates the circulation in the northern half of the model domain (Fig 7 top row) with weaker flow in the southern half of the domain. Summer shows a strongly poleward flow with high standard deviations offshore of the 2000m isobath (0.4–0.5 ms^-1^) throughout the entire domain. Autumn shows slightly weaker velocities, with a reduction in standard deviation (0.2–0.3 ms^-1^) toward the southern half of the domain. Winter shows far less variability inshore of the EAC jet (standard deviations of 0–0.15ms^-1^) with the jet still strong and separating in the northern half of the domain. The winter mean shows evidence of a recirculation / eddy dipole feature downstream of the EAC separation driving a northward flow inshore. In spring the standard deviations are again higher throughout the domain (0.25–0.4 ms^-1^) offshore of the 2000m isobath, however remain low along the coast (0–0.1 ms^-1^).

### 3.2 Seasonal temperature variability

The domain averaged time series of SST (Fig 7M) for 2012 and 2013 reveals a strong seasonal temperature cycle each year. There is a domain wide SST range of ~6°C in 2012 (min in Sept), and ~4°C in 2013 (min in July). Seasonally, temperatures were ~24°C between January and April (summer and autumn). Between April and June, temperatures were as much as ~4°C lower. Spring marked the beginning of warmer temperatures, however, the most notable distinction between 2012 and 2013 is the 3.5°C spring temperature difference between the two years.

Spatial temperature variability is shown in the surface maps of SST by season (Fig 7E–7H). Offshore and in the northern half of the domain (where velocities are strongest), mean surface temperatures were highest (up to 25°C, in Summer Fig 7E). Inshore and in the southern half of the domain, mean temperatures were lower (20–21.5°C). Standard deviations ranged from 1–2°C, with highest variability offshore (Fig 7I–7L). Along the coast mean surface temperatures were on average ~2.5°C lower in winter than summer, ranging between 18–20°C (Fig 7E and 7G). In offshore waters outside the 200 m isobath, mean temperatures were up to 25°C, in summer (which is 4–5°C higher than in winter) revealing a temperature gradient from inshore to offshore of at least 4°C (Fig 7E and 7G). Interestingly, the standard deviations were generally highest off the shelf, most likely associated with the EAC and its eddy field (presence and absence).

### 3.3 Transport estimates

**Fig 8:**
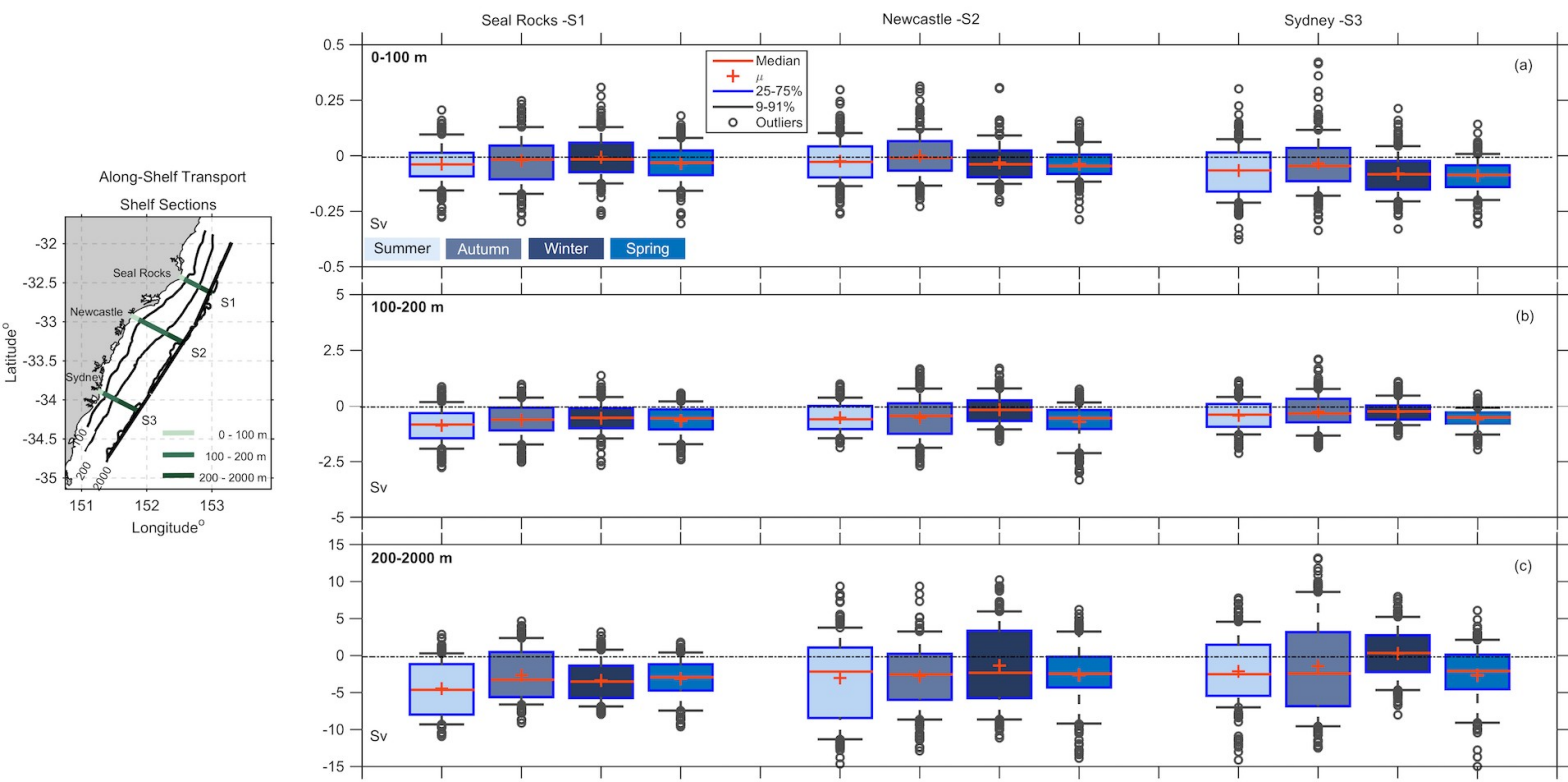
Box plots showing the along-shelf transport estimates (Sv) through three sections; Seal Rocks (S1), Newcastle (S2) and Sydney (S3), as shown in the map inset. The transports are shown across three segments a) the inner-shelf (0–100 m isobath) b) mid-shelf (0–200 m isobath), and c) across the outer shelf (200–2000 m isobath) coloured by season. Note the different scales for a, b, and c. As per the legend, the box plots show the median and the mean in red, the box shows the 25th to 75th percentiles, the tails show the 9th and 91st percentile, and the outliers are shown as circles.

To identify the major regions of shelf water import and export along the Hawkesbury Shelf and the variation with distance, we investigate transport through the 3 shore-normal sections off Seal Rocks, Newcastle and Sydney (S1, S2, S3 respectively). To compute the along-shelf transport, the sections were divided into 3 main segments representing the inner shelf (0–100 m), the middle shelf (100–200 m) and the outer shelf region (200–2000 m) as seen in (Fig 8). Width and area of each cross-shelf segment is shown in Table 1.

**Table 1:** Width (km) and area (km ^2^) of the shelf segments at three sections off Seal Rocks (S1), Newcastle (S2) and Sydney (S3).

Shelf Sections (isobaths)	S1		S2		S3	
	Seal Rocks		Newcastle		Sydney	
	(~32.4^o^S)		(~33^o^S)		(~34^o^S)	
	Width (km)	Area (km^2^)	Width (km)	Area (km^2^)	Width (km)	Area (km^2^)
0 – 100m	9.1	0.670	13.4	0.837	8.9	0.668
100 – 200m	22.2	3.070	42.1	6.522	19	3.151
200 – 2000m	16.6	18.397	25	29.155	27	28.619
0 – 2000m	48	22.137	81	36.514	55	32.438

**Fig 9:**
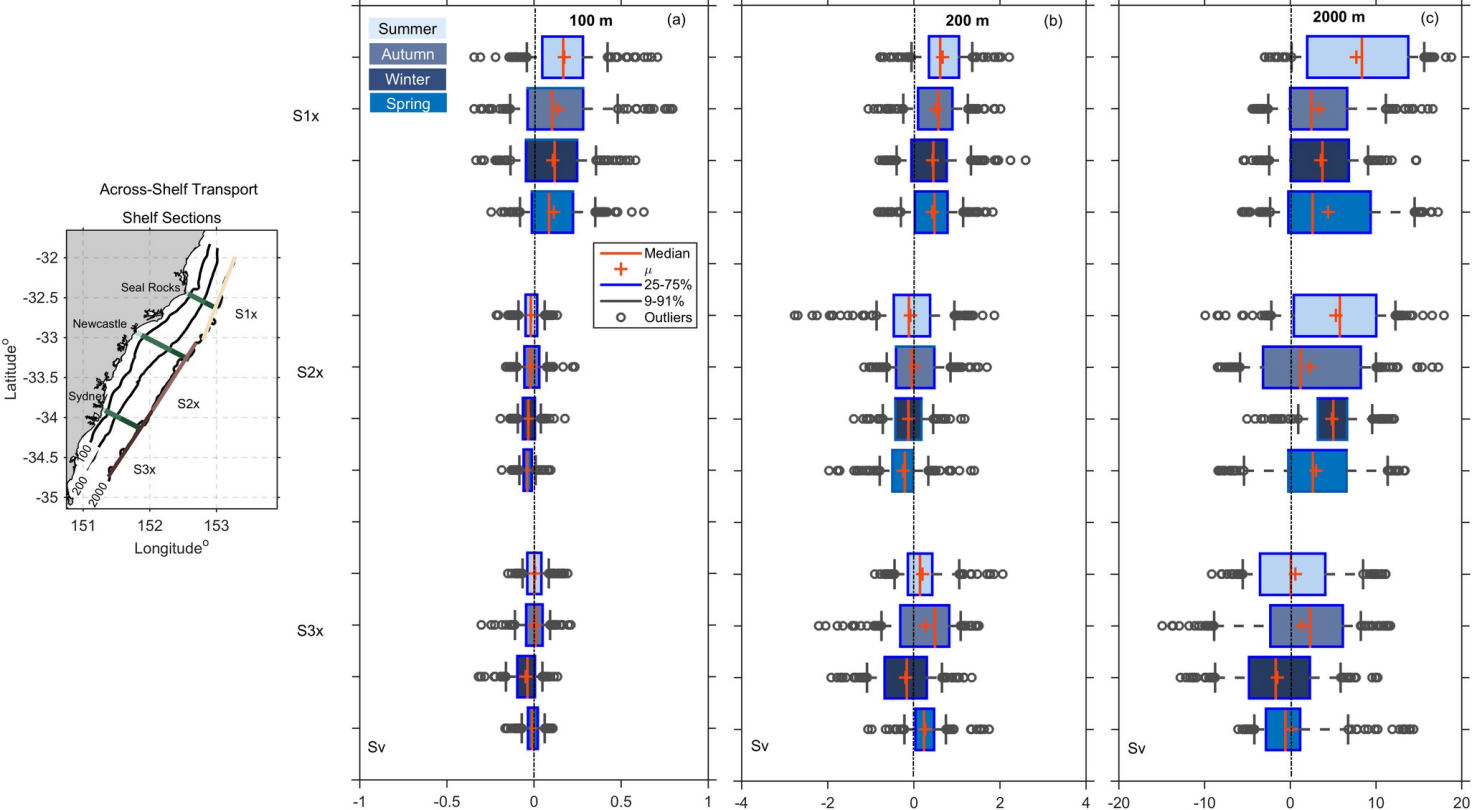
Box plots showing the cross-shelf transport estimates (in Sv) over the shelf, for 3 sections encompassing Seal Rocks (S1x), Newcastle (S2x) and Sydney (S3x) as shown in the map. Transport a) across the 100 m isobath, b) across the 200 m isobath, and c) across the 2000 m isobath for the 3 sections. Note the different scales for a, b, and c. As per the legend, the box plot shows the median, the mean, the box shows the 25th to 75th percentiles, the tails show the 9th and 91st percentile, and the outliers are shown as circles.

Cross-shelf transport was calculated through three designated along-isobath sections that span the length of the model domain i.e. transport across the inner (100m isobath), middle (200m isobath) and outer (2000m isobath) shelf. From north to south the sections for the cross-shelf transport are named S1x, S2x and S3x. S1x extends from the northern boundary and spans the Seal Rocks section (S1), S2x spans the Newcastle section (S2) and S3x spans the Sydney section (S3) and extends to the southern border (see the map inset in Fig 9). Each section is approximately 119 km long. Segments used for the cross-shelf transport calculations follow the direction of the isobath closely, even though bathymetric meanders smaller than the grid resolution (750 m) have not been accounted for in the estimates.

The normal components of velocity *v_n_* were used to compute the depth integrated across (a) and along (b) shore transports through the segments as follows:

$${(a)\;V}_{ny\pm }=\int_{-H}^{0}\int_{y_{0}}^{y_{i}}v_{n}dy\;dz;$$

$${(b)\;V}_{nx\pm }=\int_{-H}^{0}\int_{x_{0}}^{x_{i}}v_{n}dx\;dz;$$

Where the subscript + (-) denotes the offshore (onshore) transport for the cross-shore component (*V_ny_*), and equatorward (poleward) transport for the alongshore component (*V_nx_*) and *H* represents the water depth. Since daily velocity fields have been used, the free surface height was neglected. The 2-yr mean and seasonal transports and their associated standard deviations were calculated for 2012–2013 in both the along and across-shelf directions.

#### 3.3.1 Along-shelf transport

Along shelf transports are examined through 3 shelf segments along sections S1-S3 i.e. the inner (0–100 m), middle (100–200 m), and outer shelf (200–2000 m) as shown in Fig 8, and S4 Table. The results show transport is typically poleward across all sections in each season, (mean and medians). The median poleward along-shelf transport (0-2000m) off Seal Rocks (S1) is a poleward maximum during summer, median -5.3 Sv (range -9.58 –-1.59 Sv where range represents the 25–75% as per S4 Table) and is a poleward median of—3.52 Sv in Spring (range -5.92 –-1.42Sv).

Transports through the inner shelf section (0-100m) are an order of magnitude smaller than transports through 100-200m section and nearly two orders of magnitudes smaller than the 200-2000m transports. Fig 8 shows the flow is dominated by the transports at the offshore end of each section (200-2000m). Furthermore, the median transports at the offshore sections 200-2000m are strongest and least variable across the northern section (S1), whereas across the southern two sections medians are lower, but the range is more variable and greatest at the southernmost section (Figs 8C, S2 and S3).

Maximum poleward transport occurred during the summer months at each section (Fig 8, S4 Table). Through the 200-2000m section, variability was a minimum during winter and spring at S1, variability remained high through every season at S2. Off Sydney however, (S3) transport variability was greatest in autumn with high return flow although median was still poleward, (median -2.42 Sv, range -6.34–3.17 Sv). Similar values were observed off S2 during winter at the outer section. Northward flow was a maximum in winter at S2, and autumn at S3 at the offshore end of the section.

Correlations of daily along-shelf transport between the three sections, Seal Rocks, Newcastle and Sydney (as per Fig 8) were computed (Table 2) to understand if the circulation was coherent from north to south, and from onshore to offshore, or if different circulation regimes were present. Specifically, to understand the relationship between different shelf sections (i.e. to determine if the transport over the inner shelf is correlated with the middle or outer-shelf transport at each section); and to understand if the transport through the shelf sections is correlated in the along coast direction (e.g. to determine if the inner shelf transport is correlated at S1, S2 and S3). Correlations are shown in Table 2.

**Table 2:** Correlation coefficients of transport between shelf sections.

	Shelf Section	Isobath 0-100m	Isobath 100-200m	Isobath 200-2000m
(a) Along-Shelf	S1 / S2	0.65	0.37	0.63
	S1 / S3	0.55	0.04	-0.30*
	S2 / S3	0.78	0.72	0.25
	Shelf Section	Inner (0-100m) / Mid (100-200m)	Mid (100-200m) / Outer (200-2000m)	Inner (0-100m) / Outer (200-2000m)
(b) Along-Shelf	Seal Rock—S1	0.65	0.70	0.13
	Newcastle—S2	0.66	0.35	-0.03*
	Sydney—S3	0.85	0.71	0.41
	Shelf Isobath	Inner 100m / Mid 200m	Mid 200m / Outer 2000m	Inner 100m / Outer 2000m
(c) Cross-Shelf	S1x	0.60	0.47	-0.02*
	S2x	0.35	0.63	-0.04*
	S3x	0.70	0.60	0.47

(a) Correlation coefficients of along-shelf transport between shelf sections at Seal Rocks (S1) and Newcastle (S2); Seal Rocks (S1) and Sydney (S3); and Newcastle (S2) and Sydney (S3) as shown in Fig 8. (b) Correlation coefficients of along-shelf transport between shelf regions: inner (0-100m) and middle shelf (100-200m), middle (100-200m) and outer shelf (200-2000m), inner (0-100m) and outer shelf (200-2000m), at Seal Rocks (S1), Newcastle (S2) and Sydney (S3). (c) Correlation coefficients of cross-shelf transport between the 100m and 200m, 200m and 2000m, 100m and 2000m isobath sections across the long shore sections off Seal Rocks (S1x), Newcastle (S2x) and Sydney (S3x), as shown in Fig 9. Correlation coefficients shown are significant with a 99% confidence interval, unless indicated by *, not significant.

Flow through the inner shelf section (0-100m) was correlated from north to south, with cc = 0.65 correlation between Seal Rocks (S1) and Newcastle (S2), and 0.55 between Seal Rocks (S1) and Sydney (S3) and 0.78 between S2 and S3 (Table 2). At the mid-shelf (100-200m) no significant correlations were determined between Seal Rocks (S1) and Newcastle (S2), or between Seal Rocks (S1) and Sydney (S3). However, flow at Newcastle (S2) was negatively correlated with flow at Sydney (S3) with a CC = -0.72 (Table 2). At the outer shelf section (200-2000m), transport patterns off Seal Rocks (S1) and Newcastle (S2) were correlated with CC = 0.63, however the transport correlations between the other sections were low.

#### 3.3.2 Cross-shelf transport

To explore the cross-shelf transport in the region we divided the shelf into three along-shelf segments: Seal Rocks (S1x), Newcastle (S2x) and Sydney (S3x) as denoted in Fig 9. These 3 along-shelf segments span the 3 shore normal lines S1, S2, S3, respectively, that have been used throughout the previous analysis. We investigated the mean and the seasonal variability of the cross-shelf transport across the 100m, 200m and 2000m isobaths at each of the segments and the total transport from 100-2000m.

Across the northern two sections (S1x, S2x, Fig 9, S5 Table) cross-shelf transport is directed offshore in both the mean and median. Offshore transport is greatest across the 2000m isobath in the northern section (S1x) off Seal Rocks, with mean transport of 7.7 Sv (median 8.36, range 1.97–13.76 Sv where range represents the 25–75% as per S5 Table, Fig 9) directed offshore during summer. Inshore, across the 100m isobath, transports are very small (summer mean 0.17 at S1x, S5 Table, Fig 9) directed offshore.

Across the middle section, S2x, transports are more variable, but still directed offshore across the 2000m isobath in both the mean and median across all seasons (Fig 9, S5 Table), Inshore across the 100m and 200m isobaths transports are small (< 1Sv) across all seasons, directed onshore in the mean and median.

Off Sydney, (S3x), mean direction varies across the 2000m isobath, with offshore transport in summer and autumn and onshore means during winter and spring. Transport across S3x is directed onshore across all 3 isobaths isobaths during winter over this 2-yr period (2000m mean transport is -1.51Sv, median -1.7, range -4.83–2.28 Sv). Transport across the 100m isobath is directed onshore at S3x. Magnitudes are very small; less than ~0.1 Sv.

Cross-shelf transport correlations through the along-shelf sections S1x - S3x are highly variable (Table 2). Correlations are highest between the inner and mid shelf at S1x and S3x (0.6 and 0.7 respectively), whereas flows between the inner and outer shelf are uncorrelated. Cross-shelf flow between the mid and outer shelf is more correlated at S2x and S3x (0.63 and 0.6 respectively), than to the north at S1x (0.47).

### 3.4 Temporal characteristics of the transport

**Fig 10:**
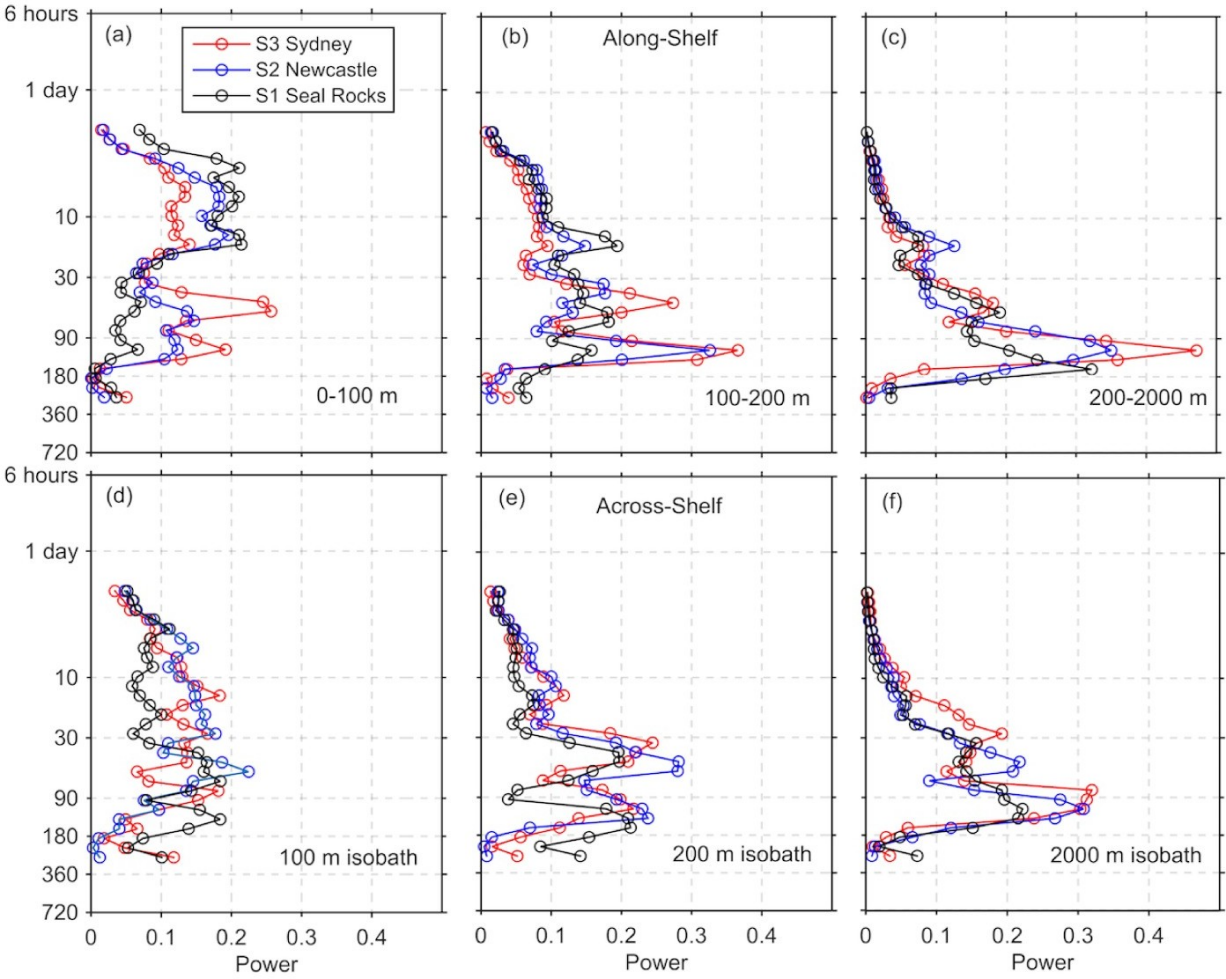
Morlet wavelet global power spectrum calculated from the daily transport timeseries, for the along-shelf transport through each of the shelf sections. (a) 0-100m, (b) 100-200m and (c) 200-2000m and for the across-shelf transport across the (d) 100m (e) 200m and (f) 2000m isobaths. Colours show the transports for each section at Seal Rocks S1, S1x, Newcastle S2, S2x, and Sydney S3, S3x in the along and cross-shelf direction, respectively.

Using wavelet analysis, we investigated the temporal periodicity and energy distribution in the along and cross-shelf transport time series (Fig 10) in order to explore possible drivers of the circulation. Although the 2-yr time series is too short to give conclusive results at low frequencies (annual and inter-annual), this analysis gives some insight at the power at higher frequencies. Energy was greatest at a periodicity of 100–140 days dominating the transport in the along-shelf direction at the mid and outer shelf regions of all three sections (S1-S3), with highest energies in the offshore region (200-2000m). Over the inner shelf (0-100m), there was increased energy at higher frequencies for the along shore transport, but not the cross-shelf transport. Highest energy at the inner shelf sections corresponded to weather band periodicities (between 8–14 days). However, energy was also observed in the 30–45-day range.

The periodicities of the cross-shelf transport (Fig 10D–10F) at the three transects had strong peaks in the mesoscale band (90–120 days), particularly across the 200m isobath. Across the 2000m isobath, power was greatest at 90 days off Sydney (S3) and 180 days to the north off Seal Rocks (S1). Cross-shelf transport energy was low in the weather band, even across the inner shelf.

### 3.5 EAC Separation from the coast

**Fig 11:**
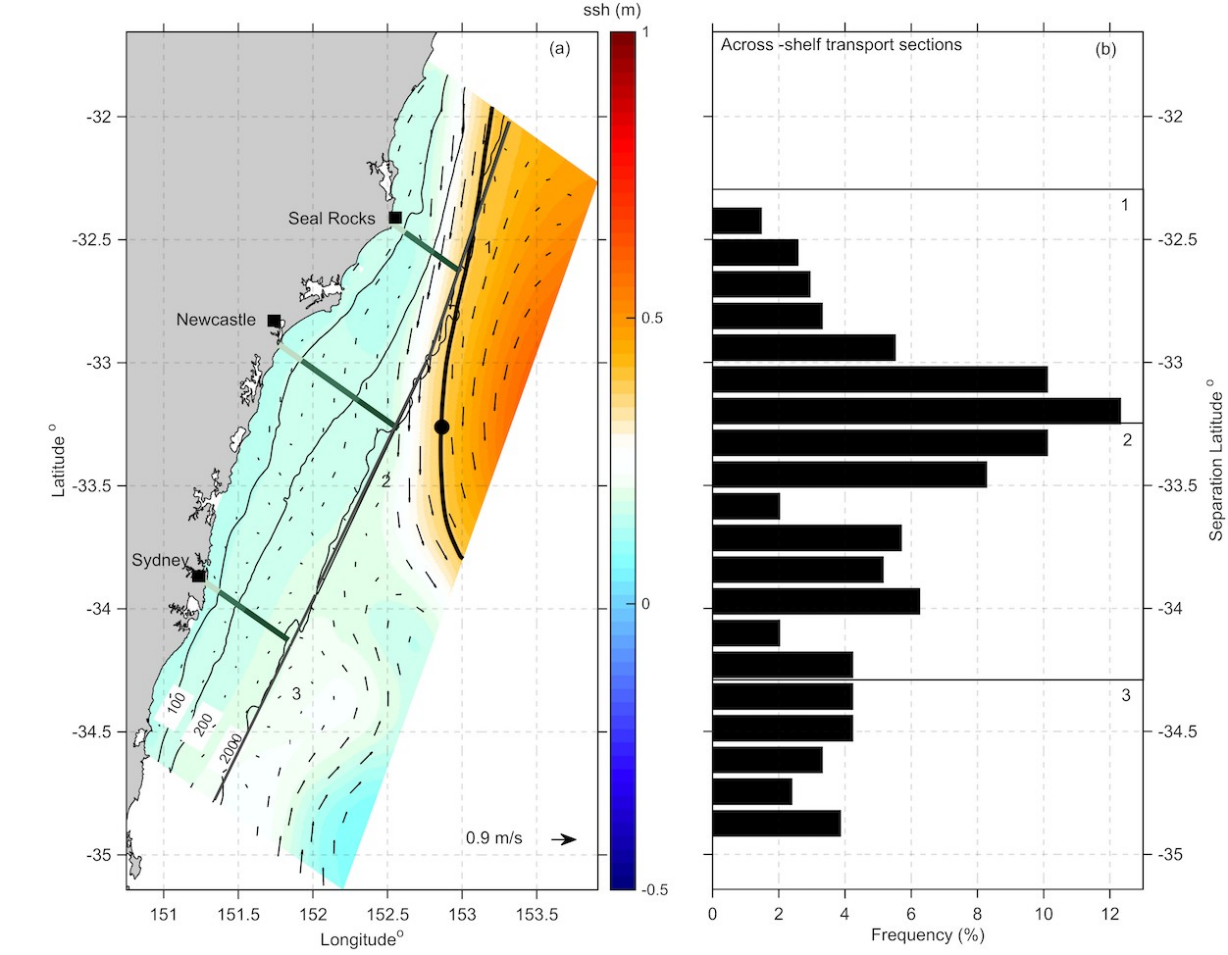
Identification of the separation latitude of the EAC. (a) Snapshot of Sea Surface Height (SSH) showing the corresponding SSH isoline used to identify the EAC separation latitude (black line and black dot respectively). Arrows present the surface flow field and solid lines show the isobath (100, 200 and 2000 m). (b) Frequency histogram of the EAC separation latitude calculated within the study domain.

To show how regions of maximum transport relate to or are influenced by the EAC separation throughout the HSM domain, the latitudinal range of the EAC separation from the coast was identified. Following the approach described fully in Cetina-Heredia et al. [1], we identify the Sea Surface Height (SSH) isoline that corresponds to the maximum poleward surface velocity across a latitudinal section at 31.5^o^S. This isoline is then followed in the poleward direction to identify the latitude at which it meanders offshore (a distance of more than 70 km from the coast). The resultant latitude is then documented as the separation latitude. This method was applied to daily-averaged velocity outputs for each day of the 2-yr simulation period and a frequency histogram was calculated by latitude (Fig 11).

The results (Fig 11) show that the EAC separates within the northern third of our domain off Seal Rocks ~35% of the time, and through S2x, off Newcastle ~40% of the time over the 2-yr period. The jet only extends down to separate off Sydney 17% of the time. The remaining time (~ 10%) the jet does not separate within our domain.

## 4. Discussion

We have presented the development and evaluation of a high resolution (750m) nested ROMS model for the Hawkesbury Shelf region of southeastern Australia and shown that the model reproduces the mean and the variability of the temperature and velocity over the shelf. The model provides a powerful tool with which to investigate coastal circulation and the alongshore and cross-shelf transport over the Hawkesbury shelf.

### 4.1 Hydrodynamic variability

The EAC is known to intensify in summer [48, 49], with an associated increase in eddy kinetic energy [4, 5]. Our model results show the intensification of the EAC in the mean surface temperature and velocity fields beyond the 200 m isobath. The highest mean temperatures ranged between 21 to 25°C (Fig 7E) and coincided with maximum poleward velocities (1.33ms^-1^) during the summer months (Fig 7A). Moreover, while the poleward flow turned eastwards between 32–33.5^o^S, a weakened and variable poleward flow with warm temperatures was present along the inner shelf. The variability of the surface circulation reflects the meandering of the EAC, with the largest variability found along the shelf-break boundary and beyond (Fig 7A–7D). This is also evident in the transport variability across the 200-2000m isobaths at each of our sections.

The cross-shelf temperature gradient is greatest in summer (up to 5°C) accentuated by upwelling driving coastal temperatures down [10, 11], and the poleward penetration of the EAC increasing the temperatures offshore. A similar temperature gradient has been observed further north at 30^o^S where a temperature gradient of up to 7°C has been measured from the coast to the core of the EAC [7, 17]. In autumn, temperatures were lower, and velocities weaker in magnitude between 32^o^S and 33.5^o^S. During spring, the pattern reversed, and the warm temperature tongue started to extend further south after being restricted to the northern domain during winter.

### 4.2 The Separation of the EAC

The EAC has been shown to separate anywhere between 28-38^o^S, but typically it separates between 31–32.5^o^S more than 50% of the time [1]. In addition, using the method of Cetina-Heredia et al. [1], Oke et al. [49] showed that typically the EAC separation location moves poleward during summer (their Fig 12) as the EAC intensifies. Our model domain extends from 32–34.5^o^S, encompassing the typical separation latitude of the EAC.

**Fig 12:**
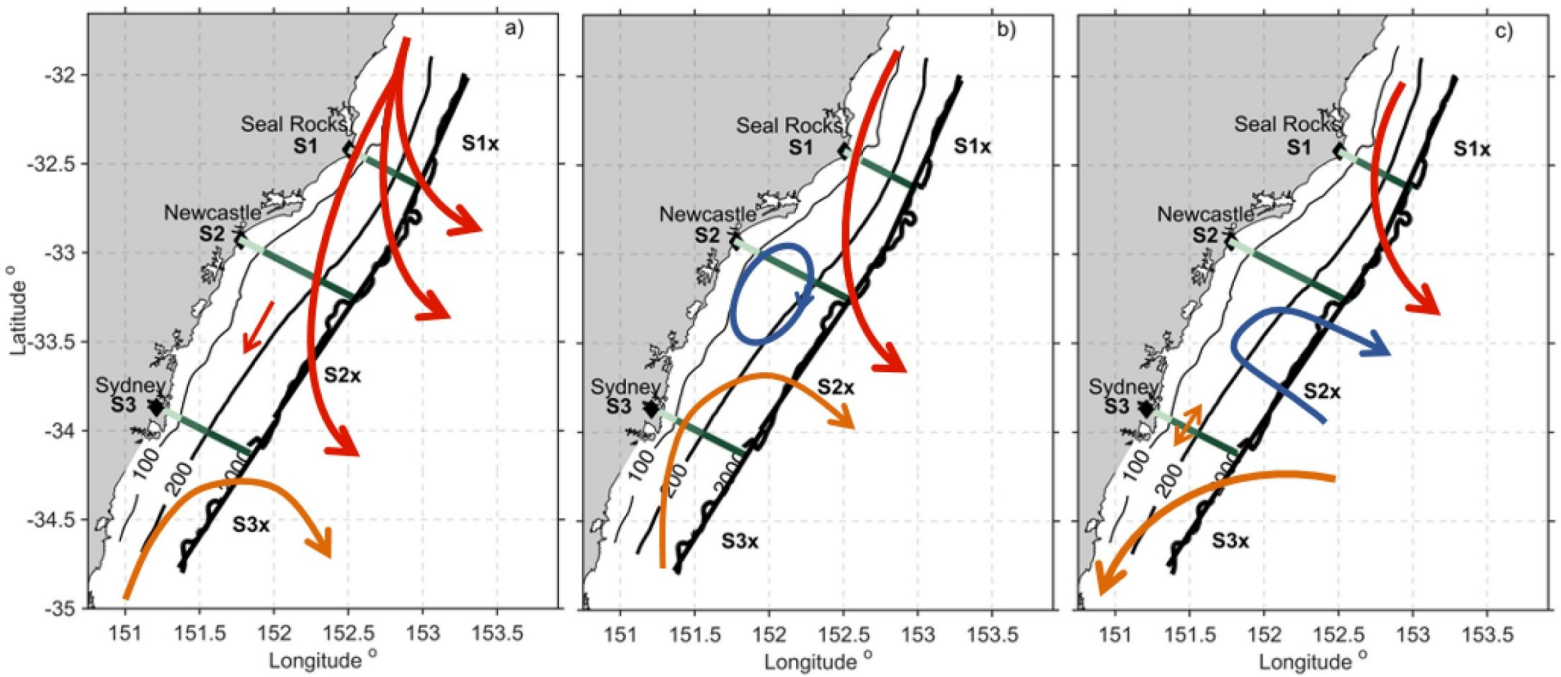
Schematic diagram representing the three main circulation patterns over the 2-yr period. (a) Scenario 1, occurring ~ 30% of the time, primarily in summer and autumn, b) Scenario 2 occurring ~20% of the time primarily in summer and autumn and c) Scenario 3 occurring ~ 11% of the time, primarily in winter and spring.

The results of the wavelet analysis (Fig 10) show a peak in the mesoscale band suggesting that the EAC is a dominant driver of the transport across the outer shelf. Furthermore, the along-shelf transports are not correlated at the offshore end of our three sections (Table 2), suggesting that our study site straddled the separation of the EAC.

To understand the impact of EAC separation as a driver of dynamics and transport in the region during our study period we investigate the variability in the separation latitude of the EAC jet using the same method of Cetina-Heredia et al. [1]. Our results (Fig 11) show that the EAC separated in the domain more than 50% of the time during our 2-yr period, and that the typical EAC separation latitude was between 33–33.5^o^S, 40% of the time. This is compared to Cetina-Heredia et al. [1], their Fig 6 who show separation peaks between 31–32.5^o^S (in a 10 km resolution model), with a lower frequency further south. The EAC separation latitude has a southward migration over the annual cycle [5], with separation typically occurring between 31.7 and 32.7^o^S with a maximum in summer. So, while the EAC separation was a little further south than normal during 2012–2013, these results give confidence that we have captured the classic ‘EAC’ separation scenario which dominates the meso scale circulation for the majority of the 2-yr period. This gives credibility to the robustness of the transport estimates shown here associated with mesoscale EAC circulation.

In the northern third of the model domain the mean geostrophic currents are dominated by the poleward flowing EAC. Maximum velocities occur where the EAC is most coherent (from 32–33^o^S), before its separation from the coast. We have shown that offshore transport on the Hawkesbury shelf is closely related to the path of the EAC and particularly with its separation, which carries water offshore across the slope/open ocean boundary (2000 m isobath) and into deeper water between 32–33.2^o^S across S1x, (Fig 9).

The consistent offshore transport across S1x (S5 Table, Fig 9) is associated with the separation of the jet which flows into the region, and then separates from the coast off Seal Rocks. Downstream of the separation point, off Sydney, cross-shelf transport is highly variable, with onshore transport dominating in autumn and winter (Fig 9, S5 Table, S3x), indicative of eddy driven transport.

Over the inner shelf, from Seal Rocks (S1) to Sydney (S3), along-shelf transport is significantly correlated (Table 2). Moreover, highest energy in the along-shelf transport on the inner-shelf occurs within the weather band, showing that the circulation on the inner shelf is likely dominated by local wind forcing.

### 4.3 Shelf exchange

The transport analysis reveals the major regions of shelf exchange. On the shelf, poleward flow dominated in the northern section off Seal Rocks (S1), but eastward flow was also a maximum through S1x. Downstream of 33^o^S, where the EAC typically separated from the coast (Figs 9 and 11), transport was more variable with a strong onshore component (Fig 9).

Most poleward transport occurs offshore close to the center of the EAC jet in the northern third of the domain (Seal Rocks, S1). In addition, the mean poleward transport in the EAC is a maximum between 31-33^o^S [5]. The northern domain encompasses the region where the EAC separates from the coast promoting offshore transport of water. Our results show that offshore export is greatest between 32-33^o^S, across the 2000m isobath. The spatial pattern and direction of the transport was consistent across seasons. The median transport across the 2000m isobath varied from 8.36 Sv during summer time to 2.45 Sv in autumn, 3.74 Sv in winter and 2.59 Sv in Spring (S5 Table, Fig 9). Noting that during summer, the offshore transport was twice as large as that during autumn and winter when the EAC inflow from the north is strongest (Fig 9).

Once the EAC has separated from the coast, poleward transport decreases in magnitude by 30% (S4 Table). Strongest onshore transport is found in the south of the domain, across S3x off Sydney (Fig 9). The cross-shelf transport was more variable downstream with transport off Sydney 33.5–34.5^o^S, varying from mean offshore in summer and autumn to mean onshore in winter and spring. The onshore transport off Sydney is driven by mesoscale EAC eddies, which are known to dominate the region downstream of the EAC separation zone [50, 51].

Long term SST data (25 years), shows the presence of a quasi-steady mesoscale eddy dipole centered at approximately 32.5^o^S [52], immediately downstream of the separation. In addition, the eddy dipole was shown to be a driver of cross-shelf transport, which is a maximum at 33-34^o^S and occurs more than 50% of the time [53]. From shipboard observations of one such event in spring 2017, it was estimated that the onshore transport (from well beyond the 2000m isobath) was be ~16Sv [53], which is within the range of our onshore transport estimates. However, ideally a longer model run would be used to better quantify the magnitude and variability of the onshore transport associated with the eddy dipole.

### 4.4 Dominant circulation patterns

To summarise, the schematic diagram in Fig 12 shows the three dominant circulation patterns that have been described here associated with the separation of the EAC and its eddy field. The EOF analysis (Fig 3) showed that mode1 occurred 34% of the time, and the transport analysis showed that it occurred primarily in summer and autumn, as represented in the schematic in Fig 12A. Mode 2 (Fig 12B) occurred 25% of the time, primarily in summer and autumn, and mode 3 (Fig 12C) occurred just 11% of the time, primarily in winter and spring. It should be noted that the direction of the flow can reverse with the passage of cyclonic and anticyclonic eddies through the domain, as reflected by the tails in transport estimate box plots (Figs 8 and 9) and the temporal expansion function of the EOFs (S3 Fig). It is the mesoscale circulation that drives shelf exchange, and shelf flows can be highly variable, associated with the different mesoscale flow patterns, driven by the location of the jet separation.

**Fig 13:**
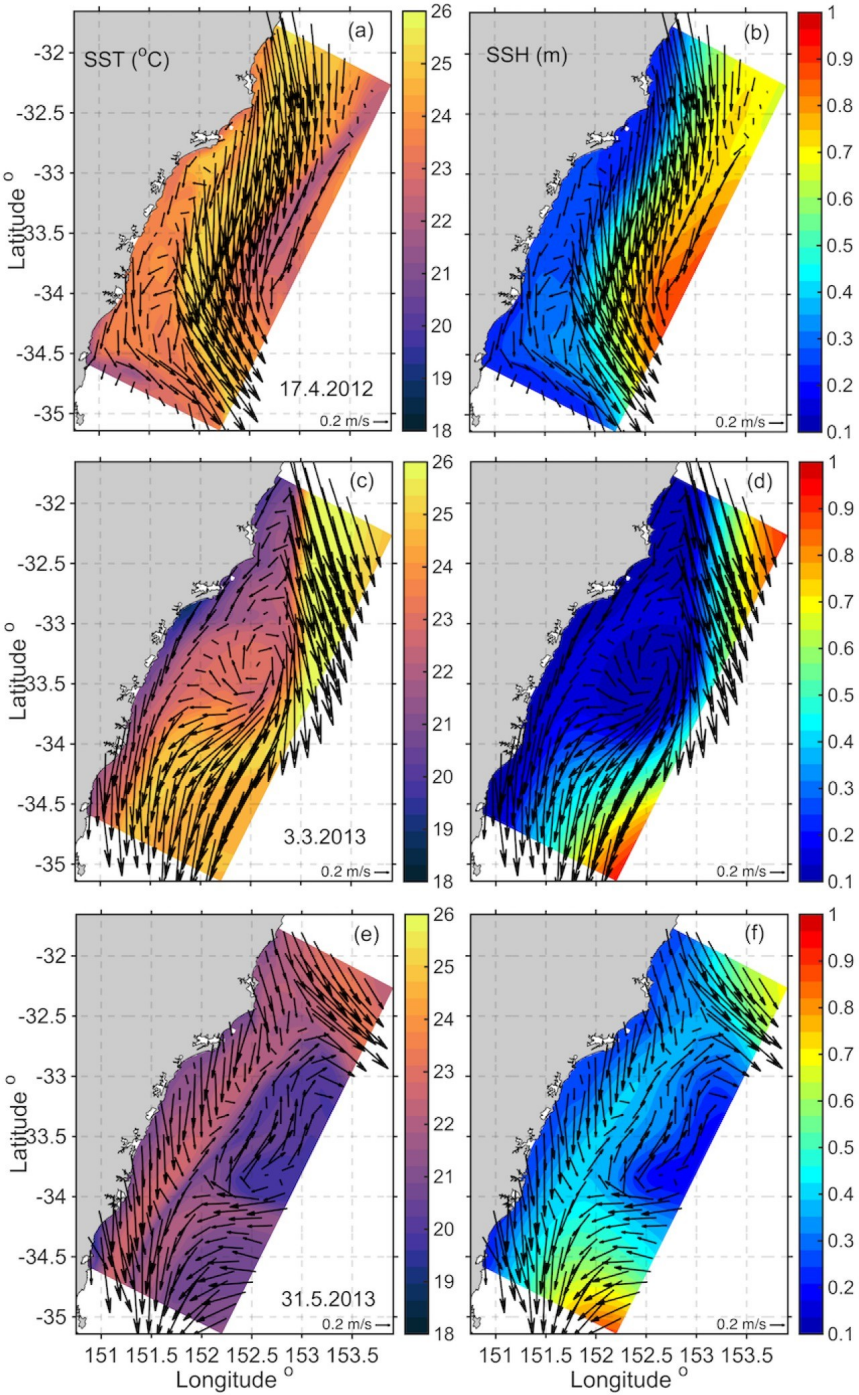
Snapshots of the three circulation scenarios shown in Fig 12. SST from the HSM model (left), and SSH with contours every 0.05m (right). Surface current vectors are shown, plotted every 25th grid cell. For (a, b) Scenario 1, 17 Apr 2012, (c, d) Scenario 2, 3 Mar 2013, and (e, f) Scenario 3, 31 May 2013.

Snapshots of the three circulation scenarios are shown in Fig 13. Scenario 1 (Fig 13A and 13B) shows the EAC dominates the domain, reflected in SST and SLA. A snapshot of scenario 2 (Fig 13C and 13D) shows lowered SSH associated with a cyclonic eddy immediately downstream of the EAC separation, with lowered sea level by the coast associated with cooler water temperatures (up to 5 degrees cooler than the core of the EAC). Also shown is the offshore flow upstream and the onshore flow in the lower third of the domain off Sydney (S3). Snapshot 3 (Fig 13E and 13F) shows a more elongated ‘pinched’ cyclonic eddy between the EAC proper (to the north) and a mesoscale anticyclonic eddy to the south with onshore flow.

### 4.5 Shelf circulation divergence and upwelling

The Hawkesbury Shelf region (Stockton Bight) has been identified as a region of persistently higher nitrate concentrations and lower temperatures (e.g. [54] their Fig 3), which Roughan and Middleton [10] attributed to EAC separation driven upwelling. Due to strong offshore transport where the EAC separates, onshore movement of water through the bottom boundary layer and up the slope may be induced downstream, resulting in upwelling events on the shelf [10]. Indeed, we see onshore transport across S2x and S3x, particularly across the 100 and 200m isobaths (Fig 9) in agreement with previous modelling studies (e.g [2] and [55]).

The snapshots in Fig 13 show examples of onshore transport, cooler SST and lower SSH by the coast associated with eddy features (Fig 13C and 13D). Upon closer inspection of the transports on the shelf in the region, divergent circulation patterns appear when counter rotating eddies dominate the region, and shelf transports can oppose each other (see Fig 12C). This scenario also appeared in the eddy dipole of Malan et al. [53] although the onshore flow associated with this particular eddy dipole feature is more poleward than the mean latitude found by Malan et al. [53]. Although their focus was on the onshore transport driven by the eddy dipole rather than the possibility of upwelling driven by alongshore divergence along the coast. This appears as another mechanism, that could contribute to upwelling and productivity in the region.

### 4.5 Limitations

As the analysis covered only a 2-yr period, care must be taken when interpreting the seasonal means and variability. As with most modelling studies, a trade off had to be made between using the most accurate boundary conditions (here we nested inside a high resolution, 2-yr data assimilating model), compared with nesting inside a free running model that has a longer time-span. Although this study was based on data from a 2-yr model run, as shown above, the results are well representative of circulation features driven by the EAC as previously observed and modelled in the region.

Due to assimilation of SST in the EAC model, temperature is resolved better in the parent model than in the HSM model. However, Figs 4 and 5 show that circulation on the shelf is resolved well by the HSM giving confidence in the use of the model velocities. The high resolution of the model allows us to make transport estimates along, on and off the shelf, that would not be possible otherwise with observations alone.

None of the 3 models discussed here (BRAN, the EAC model and the HSM model) include freshwater inflow. Generally, this is reasonable in the region because freshwater inflow is low and sporadic [41]. However, there are times such as during and immediately after sporadic east coast low events where freshwater inflow from rivers is large, resulting in salinity gradients in surface waters. The impact of this freshwater outflow on the shelf circulation is the topic of future work. Moreover, it is likely that higher resolution wind forcing will contribute to a better representation of flow on the inner shelf. Higher resolution wind fields (in time and space) would help to resolve the sea breeze and the impacts local topography driving sub-mesoscale dynamics inshore.

### 4.6 Future work

Having quantified the variability associated with seasonality in the system it is now necessary to investigate the transport associated with mesoscale dynamics of the EAC System. That is, to investigate the influence of the EAC and its eddies on along and cross-shelf transport and export from the shelf. For example, in the Gulf Stream, the cross-shelf transport at Cape Hatteras is largely driven by two converging current systems [56–58]. In the EAC we expect to see alternating periods of alongshore and cross-shelf transport depending on the mesoscale circulation (as per Fig 3). The EAC jet is known to entrain and export shelf waters offshore (containing larvae), [14, 59, 60]. Whereas Malan et al. [53] showed a persistent onshore transport associated with an eddy dipole which has been associated with onshore larval transport [1]. We can now extend this work to look at the transport associated with each of these scenarios.

While this study has not focused on sub-mesoscale processes, now that the model has been shown to perform well in the region, it could be used to investigate sub-mesoscale processes. Further north, off Coffs Harbour (30° S), Kerry et al. [61] showed that a 750m resolution model provided improved representation of sub-mesoscale features inshore of the EAC (compared to the coarser EAC model). In this region, Archer et al. [52] identified sub-mesoscale processes including frontogenesis and instabilities associated with the separation of the EAC jet and high strain between two counter rotating eddies. The HSM model could now be used to explore the dynamics of such features more fully and their role in vertical and horizontal exchange. Alternatively, the HSM could be used as boundary conditions for an even higher resolution model with which to explore sub-mesoscale dynamics.

Finally, as this is known to be a highly productive region (in what is a typically oligotrophic WBC environment) the model is now being used to understand fine scale circulation on the shelf, and the role of the connectivity of larval populations onto and along the shelf in what is known to be a highly productive region. In terms of model development, future work will include extending the model for a multi decadal time period, with the inclusion of freshwater and coastal winds.

## 5. Conclusion

A high resolution (750 m) numerical model has been configured and validated for the Hawkesbury Shelf (2012–2013). Comparisons of modelled and observed temperature and velocities confirm that the HSM reproduces the dominant dynamical features of the mesoscale and coastal variability in the domain.

The temperature in the region varies seasonally. The overall mean circulation is dominated by the poleward flowing EAC with maximum velocities found along the shelf break. Cross-shelf velocities are an order of magnitude weaker than along-shelf flows. Downstream of the separation, a meandering EAC and the frequent encroachments of eddies lead to a much more variable flow pattern offshore of Sydney and Newcastle. The inner shelf regions (between the coast and the 100m isobath) however, are less influenced by the EAC with highest energy in the weather band.

The mean cross-shelf transport across the 2000 m isobath (i.e. the shelf/slope boundary) shows that the region between 32^o^S– 33^o^S (encompassing Seal Rocks) is the major site for shelf water export into the open ocean with median seasonal offshore transports ranging ~– 2.5–8.4 Sv across the 2000m isobath and is a maximum in summer. Further south, off Newcastle and Sydney, the cross-shore transport is highly variable with standard deviations generally larger than the mean. Onshore transport occurs more frequently off Sydney 33.5–34.5^o^S; seasonal medians range -1.7 to 2.3Sv across the 2000m isobath, with an onshore maximum in winter. Transport is generally driven by the mesoscale EAC circulation, and its separation from the coast, and the mesoscale eddy field downstream of the EAC separation point. The development of this model allows its use in future studies at high resolution, including investigating drivers of productivity on the Hawkesbury Shelf, in what is an oligotrophic western boundary current regime.

## Supporting information

S1 Fig Snapshot of domain wide sea surface temperature from 3 model resolutions for the 9 of January 2013: (a) Bluelink Reanalysis 10 km x 50 z-levels, (b) EAC model ~2.5–6 km x 30 sigma level, (c) HSM 0.75 km x 30 sigma levels. Black boxes show the boundaries of the zoomed region below (d-f). Solid black lines indicate the 100 and 200 m isobaths, respectively. Vertical sections are shown in (g-i).
(TIF)

S2 Fig (a) Zoom of the bathymetry showing the southern boundary of the Hawkesbury Shelf model (HSM) and demonstrating the matching of the EAC model bathymetry to that of the HSM boundary region. HSM isobaths are shown in white, EAC model isobaths are shown in black. (b) Schematic showing the improved grid cell resolution along the southern boundary of the Hawkesbury Shelf Model (HSM).
(TIF)

S3 Fig The principal component time series (PC) for EOF modes. (a) Mode 1, (b) Mode 2, (c) Mode 3 from the HSM (blue) and observations (red), respectively. CC stands for the correlation coefficient between the HSM and observations. The correlation coefficients are above the 95% level of confidence.
(TIF)

S4 Fig **Sea level height (meters) simulated by HSM (blue) and tide gauge data at Fort Denison (red).** (CC = 0.93, with 95% confidence level) between shelf model and tide gauge. (a): 48-day time series. (b) 18-day zoom. The mean has been removed from both series. CC is the correlation between the observed and simulated (HSM) time series. Both the model and tide gauge data are in UTC time.
(TIF)

S5 Fig **Rotary spectra for depth-averaged hourly velocities (left column: Clockwise, right column: Counter-clockwise) for modelled (HSM, blue) and observed (red) velocities (moorings off Sydney, ORS065, SYD100, SYD140).** The dot-dash lines show tidal period in the diurnal (D) and semi-diurnal (S) band and the dashed line shows the inertial period (I = 21.4 hrs).
(TIF)

S6 Fig **Taylor diagram showing correlations, standard deviations and RMS errors between the model and the observations (from HSM in blue and EAC model in red) vs observations (temperature loggers).** The radial coordinate of the Taylor diagram is the normalized standard deviation with respect to observations, the angular coordinate is the Pearson correlation and grey arcs represent centered root mean square difference between the models and the observations. Circles: SYD140, triangle: SYD100, square: ORS065.
(TIF)

S1 Table Location and depth information (sensor and observation depth intervals) of the NSW mooring array off Sydney (Roughan and Morris [25]). Mooring data for SYD140 and ORS065 are available from Jan 2012-Dec 2013, SYD100 from Sept 2012-Dec 2013.
(DOCX)

S2 Table Comparison of tidal amplitudes and phases (relative to Greenwich) between the HSM and sea level height observations at the tide gauge Fort Denison in Sydney Harbour (Fig 1) for the 6 main tidal constituents as shown in the table. Also shown are the RMS errors between the model and the data.
(DOCX)

S3 Table Mean depth-averaged current speeds for the across-shelf (U), and along-shelf (V) velocities and their associated axis orientations for variance ellipses: (Top) upper—depth bin, (centre) mid—depth bin, (bottom) bottom—depth bin.
(DOCX)

S4 Table Table showing mean, median, standard deviation, 25^th^ and 75^th^ percentiles of along-shelf transport (in Sv) for each season through shelf transects off Seal Rocks (S1), Newcastle (S2) and Sydney (S3) on the Hawkesbury Shelf. Shelf transects represent the inner shelf (0-100m), middle shelf (100-200m), outer-shelf (200-2000m) and the entire shelf (0–2000 m).
(DOCX)

S5 Table Table showing mean, median, standard deviation, 25^th^ and 75^th^ percentiles of across-shore transport (in Sv) for each season through and each season through three along shelf transects off Seal Rocks (S1x), Newcastle (S2x) and Sydney (S3x) on the Hawkesbury Shelf across 3 isobaths, 100m, 200m and 2000m and the total 100-2000m. (DOCX)
