# Supplementary figures and images for: Transport variability over the Hawkesbury Shelf (31.5–34.5°S) driven by the East Australian Current
Source: PLoS One. 2020 Nov 5;15(11):e0241622. doi: 10.1371/journal.pone.0241622 (PMC7644073; doi:10.1371/journal.pone.0241622)

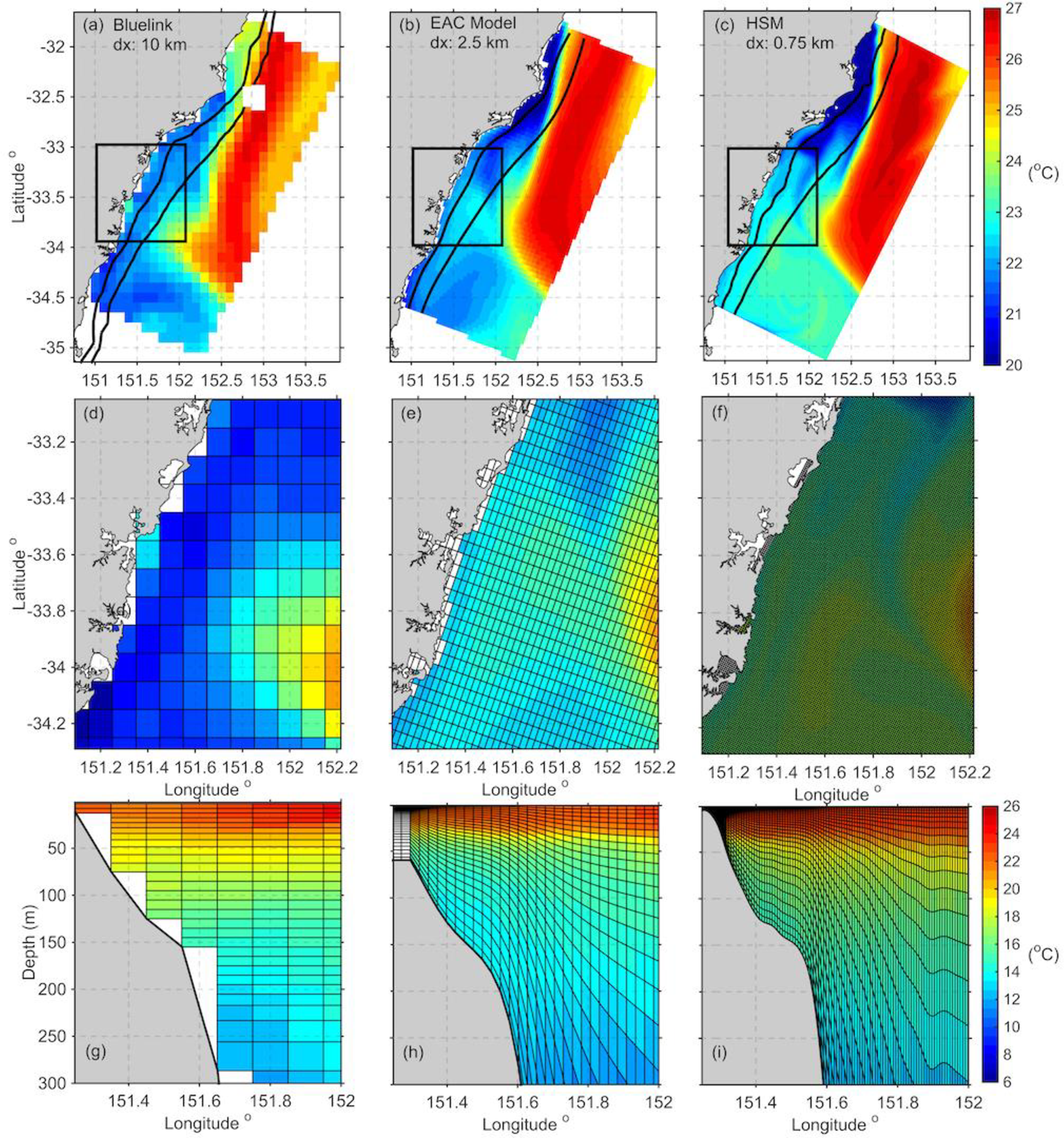

Supplement: S1 Fig — Snapshot of domain wide sea surface temperature from 3 model resolutions for the 9 of January 2013: (a) Bluelink Reanalysis 10 km x 50 z-levels, (b) EAC model ~2.5–6 km x 30 sigma level, (c) HSM 0.75 km x 30 sigma levels. Black boxes show the boundaries of the zoomed region below (d-f). Solid black lines indicate the 100 and 200 m isobaths, respectively. Vertical sections are shown in (g-i). (TIF) [file pone.0241622.s001.tif]

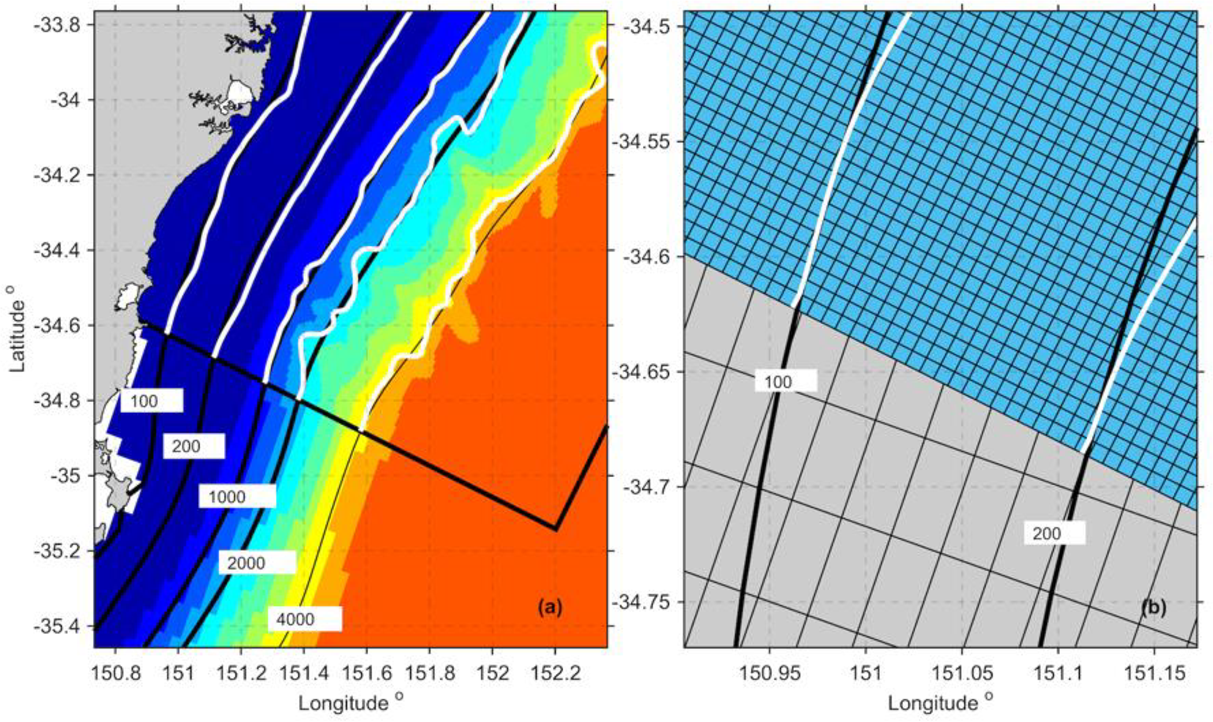

Supplement: S2 Fig — (a) Zoom of the bathymetry showing the southern boundary of the Hawkesbury Shelf model (HSM) and demonstrating the matching of the EAC model bathymetry to that of the HSM boundary region. HSM isobaths are shown in white, EAC model isobaths are shown in black. (b) Schematic showing the improved grid cell resolution along the southern boundary of the Hawkesbury Shelf Model (HSM). (TIF) [file pone.0241622.s002.tif]

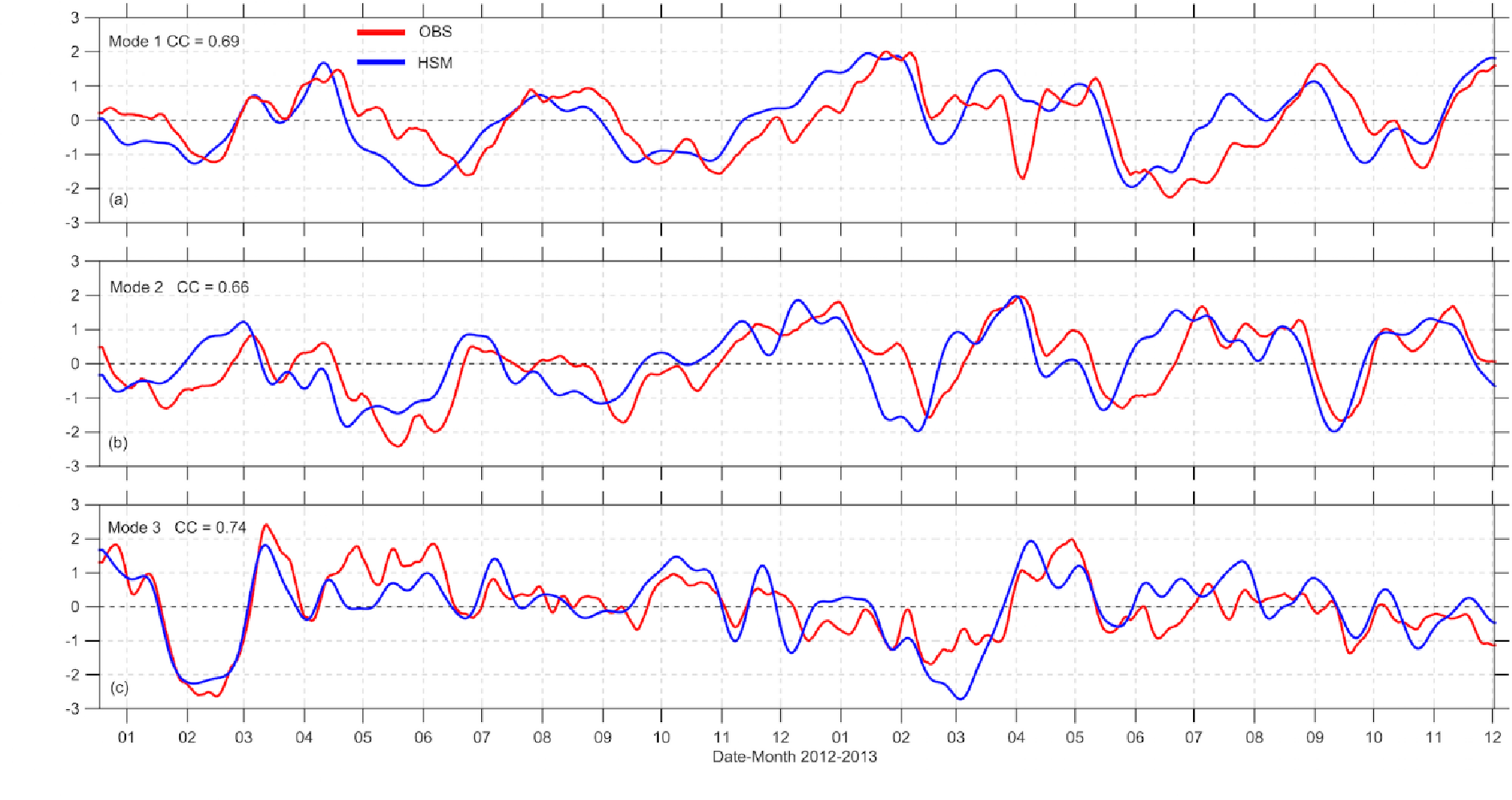

Supplement: S3 Fig — (a) Mode 1, (b) Mode 2, (c) Mode 3 from the HSM (blue) and observations (red), respectively. CC stands for the correlation coefficient between the HSM and observations. The correlation coefficients are above the 95% level of confidence. (TIF) [file pone.0241622.s003.tif]

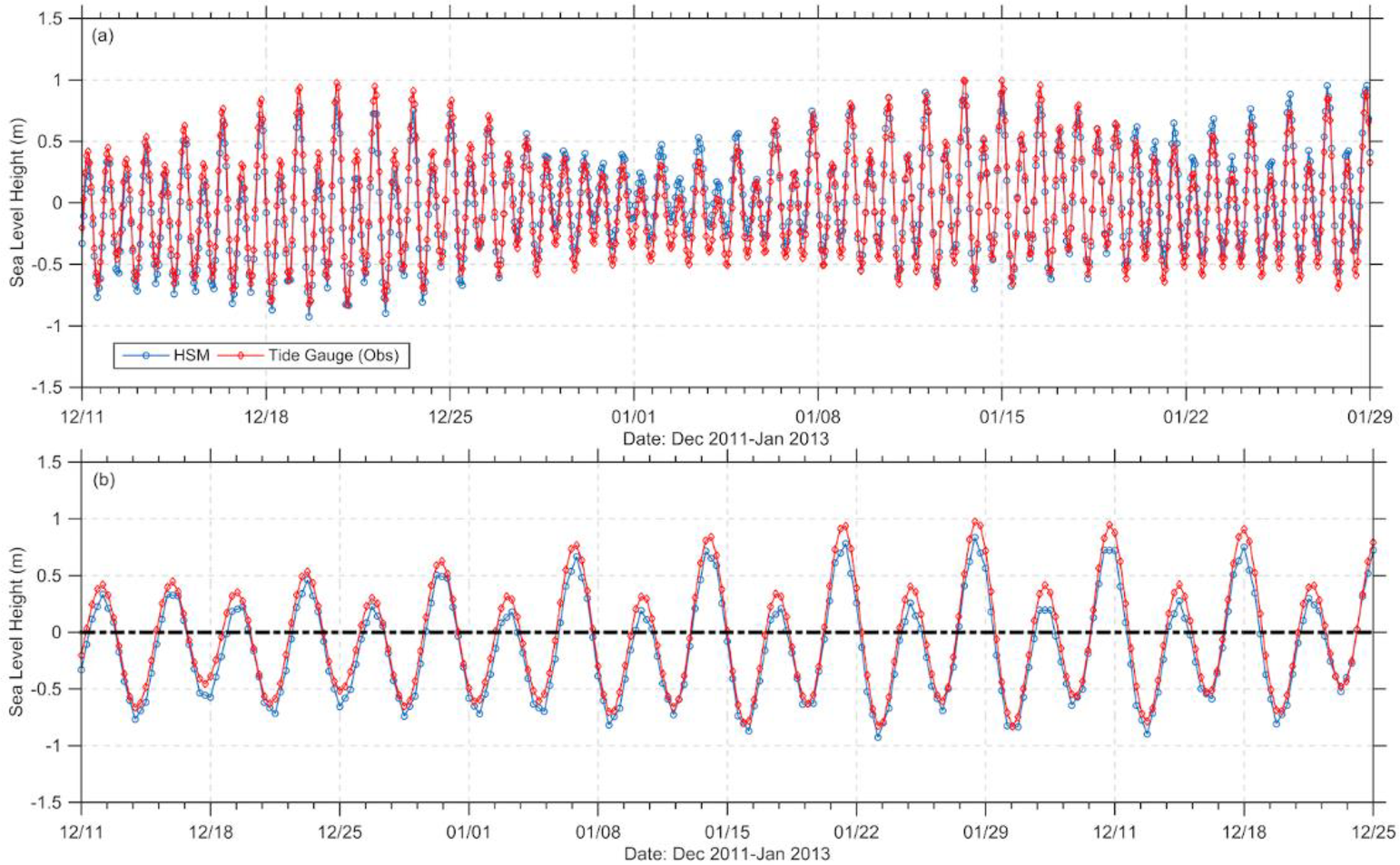

Supplement: S4 Fig — Sea level height (meters) simulated by HSM (blue) and tide gauge data at Fort Denison (red). (CC = 0.93, with 95% confidence level) between shelf model and tide gauge. (a): 48-day time series. (b) 18-day zoom. The mean has been removed from both series. CC is the correlation between the observed and simulated (HSM) time series. Both the model and tide gauge data are in UTC time. (TIF) [file pone.0241622.s004.tif]

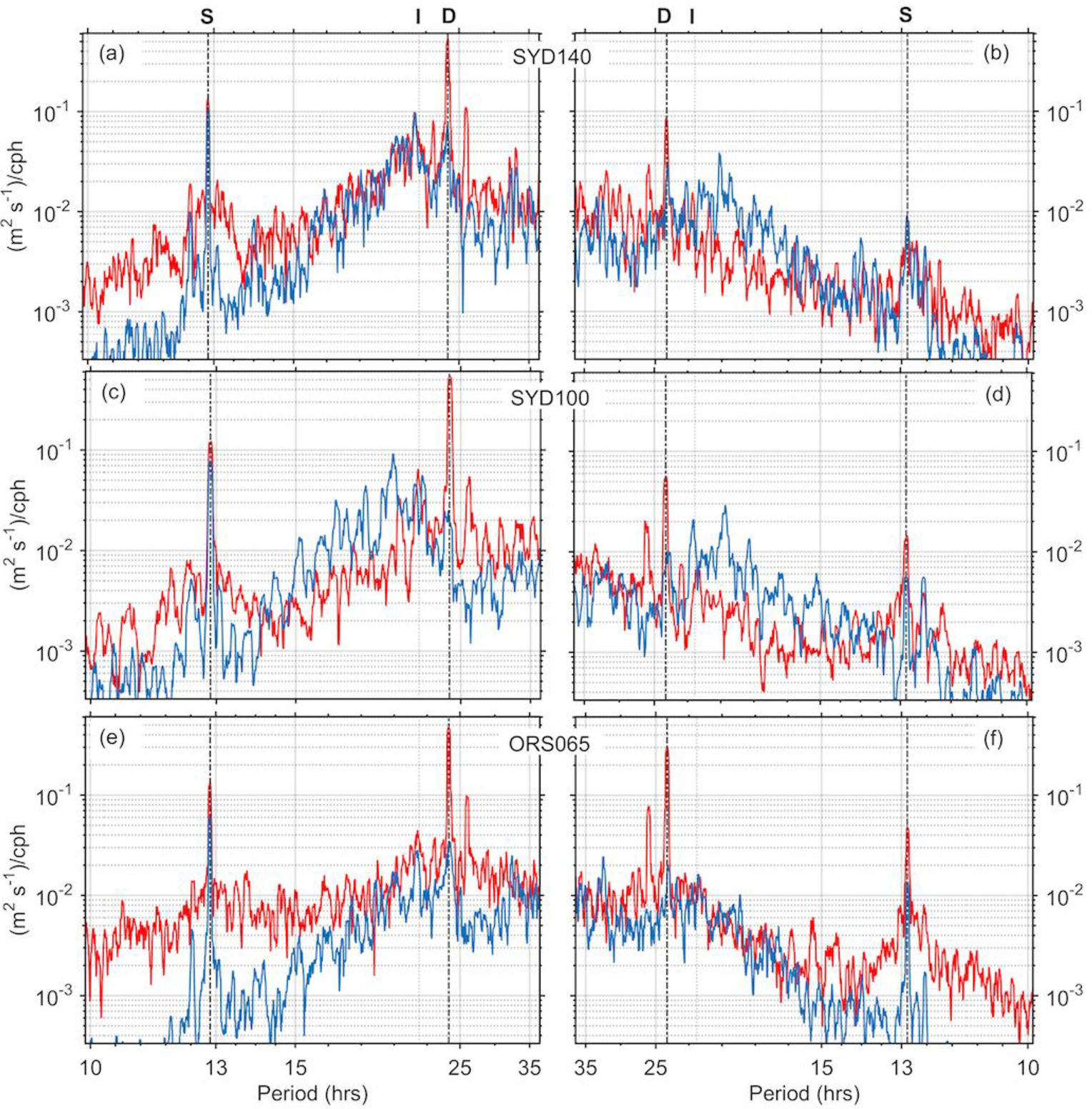

Supplement: S5 Fig — Rotary spectra for depth-averaged hourly velocities (left column: Clockwise, right column: Counter-clockwise) for modelled (HSM, blue) and observed (red) velocities (moorings off Sydney, ORS065, SYD100, SYD140). The dot-dash lines show tidal period in the diurnal (D) and semi-diurnal (S) band and the dashed line shows the inertial period (I = 21.4 hrs). (TIF) [file pone.0241622.s005.tif]

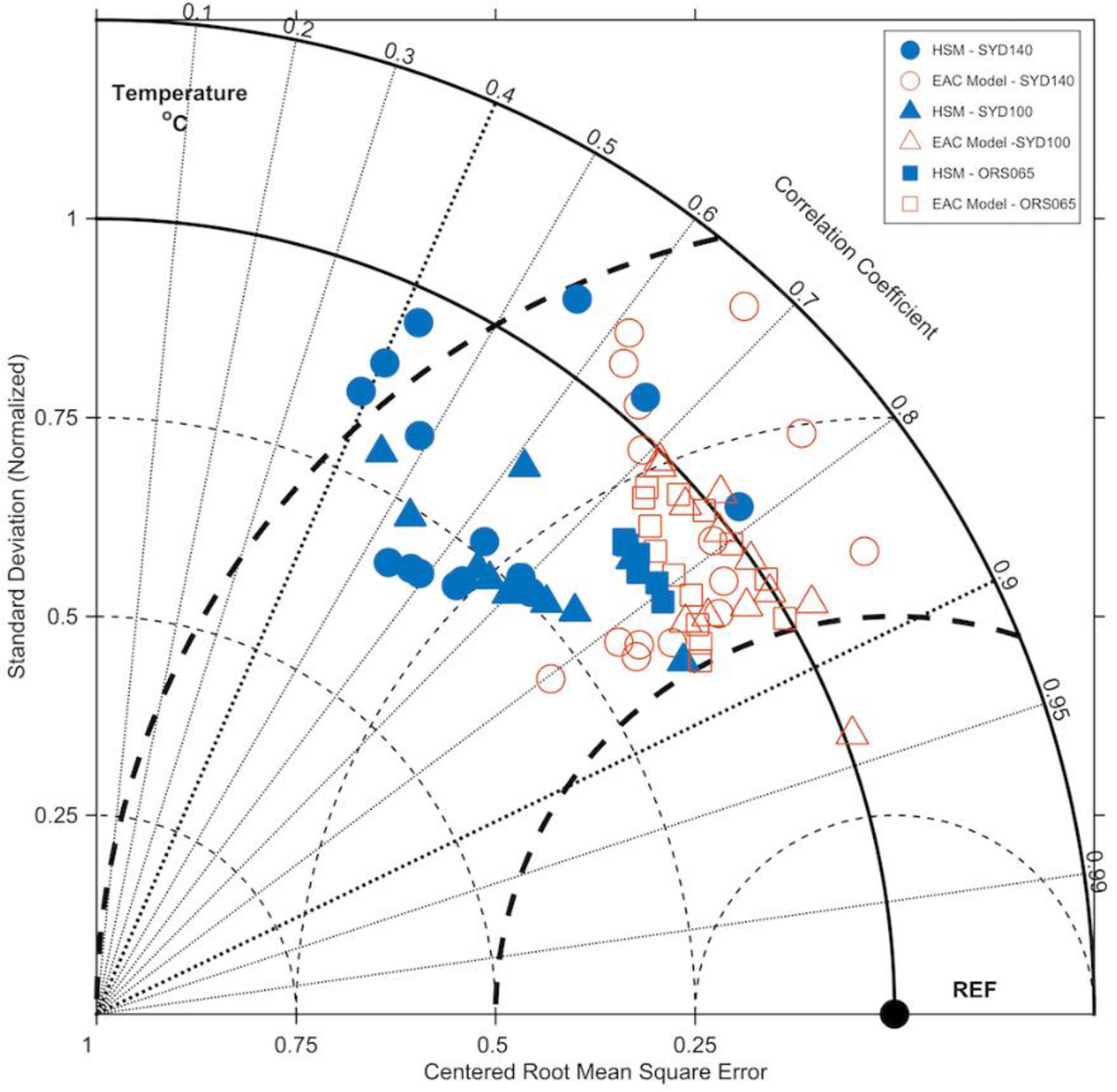

Supplement: S6 Fig — Taylor diagram showing correlations, standard deviations and RMS errors between the model and the observations (from HSM in blue and EAC model in red) vs observations (temperature loggers). The radial coordinate of the Taylor diagram is the normalized standard deviation with respect to observations, the angular coordinate is the Pearson correlation and grey arcs represent centered root mean square difference between the models and the observations. Circles: SYD140, triangle: SYD100, square: ORS065. (TIF) [file pone.0241622.s006.tif]
