# Supplementary material for: Transport variability over the Hawkesbury Shelf (31.5–34.5°S) driven by the East Australian Current
Source: PLoS One. 2020 Nov 5;15(11):e0241622. doi: 10.1371/journal.pone.0241622 (PMC7644073; doi:10.1371/journal.pone.0241622)
Supplement: S1 Table — Mooring data for SYD140 and ORS065 are available from Jan 2012-Dec 2013, SYD100 from Sept 2012-Dec 2013. (DOCX) [file pone.0241622.s007.docx]

| Mooring Name | Location | | Water Depth | Distance from Shore | Observation Depth Intervals | | Sensor Depth Range | |
| --- | --- | --- | --- | --- | --- | --- | --- | --- |
|  |  |  |  |  | ADCP | Temp | ADCP | Temp |
|  | Lat (^o^ S) | Long (^o^ E) | (m) | (km) | (m) | (m) | (m) | (m) |
| ORS065 | 33.8975 | 151.3153 | 67 | 2 | 2 | 4 | 7-59 | 16-67 |
| SYD100 | 33.9429 | 151.3821 | 104 | 10 | 4 | 8 | 21-97 | 18-104 |
| SYD140 | 33.9944 | 151.4588 | 138 | 19 | 8 | 8 | 39-127 | 26-143 |

S1 Table: Location and depth information (sensor and observation depth intervals) of the NSW Mooring array off Sydney (Roughan and Morris (2011)). Mooring data for SYD140 and ORS065 are available from Jan 2012-Dec 2013, SYD100 from Sept 2012-Dec 2013.

Citation

Roughan, M., Morris, B., 2011. Using high-resolution ocean timeseries data to give context to long term hydrographic sampling off Port Hacking, NSW, Australia, OCEANS'11-MTS/IEEE.IEEE, Piscataway, NJ, United States., Kona, Hawaii,1–4.
