# Supplementary material for: Transport variability over the Hawkesbury Shelf (31.5–34.5°S) driven by the East Australian Current
Source: PLoS One. 2020 Nov 5;15(11):e0241622. doi: 10.1371/journal.pone.0241622 (PMC7644073; doi:10.1371/journal.pone.0241622)
Supplement: S2 Table — Also shown are the RMS errors between the model and the data. (DOCX) [file pone.0241622.s008.docx]

| Tidal  Constituents | | Amplitude  (m) | Phase  (^o^) | RMS  (m) | RMS  (%) |
| --- | --- | --- | --- | --- | --- |
| M2 | Tide Gauge  HSM | 0.51  0.47 | 305.98  305.12 | 0.028 | 5.55 |
| K1 | Tide Gauge  HSM | 0.16  0.15 | 329.27  314.91 | 0.057 | 38.00 |
| S2 | Tide Gauge  HSM | 0.13  0.11 | 332.19  320.91 | 0.021 | 16.15 |
| N2 | Tide Gauge  HSM | 0.11  0.11 | 298.82  297.20 | 0.006 | 5.55 |
| O1 | Tide Gauge  HSM | 0.09  0.09 | 312.31  305.35 | 0.008 | 8.89 |
| Q1 | Tide Gauge  HSM | 0.02  0.02 | 287.98  285.99 | 0.001 | 5.00 |

S2 Table: Comparison of tidal amplitudes and phases (relative to Greenwich) between the HSM and sea level height observations at the tide gauge Fort Denison in Sydney Harbour (Figure 1) for the 6 main tidal constituents as shown in the table. Also shown are the RMS errors between the model and the data.
