# Supplementary material for: Transport variability over the Hawkesbury Shelf (31.5–34.5°S) driven by the East Australian Current
Source: PLoS One. 2020 Nov 5;15(11):e0241622. doi: 10.1371/journal.pone.0241622 (PMC7644073; doi:10.1371/journal.pone.0241622)
Supplement: S3 Table — Mean depth-averaged current speeds for the across-shelf (U), and along-shelf (V) velocities and their associated axis orientations for variance ellipses: (Top) upper—depth bin, (centre) mid—depth bin, (bottom) bottom—depth bin. (DOCX) [file pone.0241622.s009.docx]

|  |  |  | ORS65 | SYD100 | SYD140 |
| --- | --- | --- | --- | --- | --- |
| Upper | U Across-shelf (m s^-1^) | OBS  HSM | -0.034  -0.034 | -0.049  -0.042 | -0.073  -0.055 |
|  | V Across-shelf (m s^-1^) | OBS  HSM | 12.89  10.74 | -0.114  -0.149 | -0.163  -0.128 |
|  | Major axis orientation (^o^) | OBS  HSM | 12.89  10.74 | 21.04  19.27 | 24.16  24.23 |
|  | Minor axis orientation (^o^) | OBS  HSM | 0.03  0.04 | 0.06  0.04 | 0.08  0.04 |
|  |  |  |  |  |  |
| Middle | U Across-shelf (m s^-1^) | OBS  HSM | -0.017  -0.023 | -0.036  -0.036 | -0.043  -0.059 |
|  | V Across-shelf (m s^-1^) | OBS  HSM | -0.062  -0.117 | -0.075  -0.129 | -0.110  -0.133 |
|  | Major axis orientation (^o^) | OBS  HSM | 13.25  11.60 | 18.62  19.38 | 23.08  24.45 |
|  | Minor axis orientation (^o^) | OBS  HSM | 0.03  0.02 | 0.05  0.03 | 0.06  0.04 |
|  |  |  |  |  |  |
| Bottom | U Across-shelf (m s^-1^) | OBS  HSM | -0.016  -0.007 | -0.000  -0.021 | -0.017  -0.038 |
|  | V Across-shelf (m s^-1^) | OBS  HSM | -0.036  -0.072 | -0.029  -0.091 | -0.060  -0.096 |
|  | Major axis orientation (^o^) | OBS  HSM | 21.00  16.62 | 19.98  17.55 | 28.24  24.71 |
|  | Minor axis orientation (^o^) | OBS  HSM | 0.02  0.02 | 0.04  0.02 | 0.05  0.03 |

S3 Table: Mean depth-averaged current speeds for the across-shelf (U), and along-shelf (V) velocities and their associated axis orientations for variance ellipses: (top) upper - depth bin, (centre) mid - depth bin, (bottom) bottom - depth bin.
