# Supplementary material for: Transport variability over the Hawkesbury Shelf (31.5–34.5°S) driven by the East Australian Current
Source: PLoS One. 2020 Nov 5;15(11):e0241622. doi: 10.1371/journal.pone.0241622 (PMC7644073; doi:10.1371/journal.pone.0241622)
Supplement: S4 Table — Shelf transects represent the inner shelf (0-100m), middle shelf (100-200m), outer-shelf (200-2000m) and the entire shelf (0–2000 m). (DOCX) [file pone.0241622.s010.docx]

|  |  | Summer | | | | | Autumn | | | | | Winter | | | | | Spring | | | | |
| --- | --- | --- | --- | --- | --- | --- | --- | --- | --- | --- | --- | --- | --- | --- | --- | --- | --- | --- | --- | --- | --- |
|  | Shelf-Section | Mean | Median | 25 % | 75 % | SD | Mean | Median | 25 % | 75 % | SD | Mean | Median | 25 % | 75 % | SD | Mean | Median | 25 % | 75 % | SD |
| S1 | 0-100m | 0 | -0.04 | -0.09 | 0.01 | 0.10 | 0 | -0.02 | -0.11 | 0.05 | 0.10 | 0 | -0.03 | -0.07 | 0.06 | 0.10 | -0.03 | -0.03 | -0.09 | 0.02 | 0.10 |
|  | 100-200m | -0.90 | -0.83 | -1.45 | -0.32 | 0.80 | -0.60 | -0.62 | -1.00 | -0.07 | 0.80 | -0.60 | -0.53 | -1.00 | -0.09 | 0.70 | -0.65 | -0.55 | -1.04 | -0.15 | 0.70 |
|  | 200-2000m | -4.50 | -4.63 | -7.98 | -1.67 | 3.70 | -2.70 | -3.27 | -6.62 | 0.48 | 3.40 | -3.40 | -3.51 | -5.74 | -1.38 | 2.80 | -3.12 | -2.94 | -4.73 | 1.18 | 2.70 |
|  | 0-2000m | -5.40 | -5.30 | -9.58 | -1.59 | 4.30 | -3.20 | -3.91 | -6.48 | -0.24 | 3.90 | -3.90 | -3.88 | -6.55 | -1.43 | 3.30 | -3.80 | -3.52 | -5.92 | 1.42 | 3.20 |
|  |  |  |  |  |  |  |  |  |  |  |  |  |  |  |  |  |  |  |  |  |  |
| S2 | 0-100m | 0 | -0.03 | -0.09 | 0.04 | 0.10 | 0 | -0.01 | -0.07 | 0.07 | 0.10 | 0 | -0.04 | -0.09 | 0.02 | 0.10 | 0.04 | -0.05 | -0.08 | 0.01 | 0.10 |
|  | 100-200m | -0.50 | -0.60 | -1.45 | 0 | 0.60 | -0.50 | -0.45 | -1.25 | 0.12 | 1.00 | -0.20 | -0.18 | -0.67 | 0.25 | 0.70 | -0.72 | -0.55 | -1.04 | -0.18 | 0.90 |
|  | 200-2000m | -3.10 | -2.17 | -8.45 | 1.07 | 5.70 | -2.80 | -2.80 | -5.99 | 0.24 | 4.20 | -1.40 | -2.34 | -5.76 | 3.36 | 5.40 | -2.62 | -2.47 | -4.32 | -0.16 | 4.10 |
|  | 0-2000m | -3.50 | -2.80 | -8.96 | 0.37 | 5.80 | -3.30 | -3.26 | -6.23 | -0.14 | 4.70 | -1.60 | -1.81 | -6.19 | 2.83 | 5.70 | -3.37 | -3.33 | -5.18 | -0.42 | 4.70 |
|  |  |  |  |  |  |  |  |  |  |  |  |  |  |  |  |  |  |  |  |  |  |
| S3 | 0-100m | -0.10 | -0.07 | -0.16 | 0.02 | 0.10 | 0 | -0.05 | -0.11 | 0.04 | 0.10 | -0.10 | -0.08 | -0.15 | -0.02 | 0.10 | -0.09 | -0.09 | -0.14 | -0.04 | 0.10 |
|  | 100-200m | -0.40 | -0.39 | -0.93 | 0.09 | 0.70 | -0.20 | -0.33 | -0.73 | 0.32 | 0.80 | -0.30 | -0.25 | -0.86 | 0.02 | 0.50 | -0.58 | -1.29 | -0.79 | -0.29 | 0.50 |
|  | 200-2000m | -2.10 | -2.52 | -5.45 | 1.46 | 4.70 | -1.50 | -2.42 | -6.34 | 3.17 | 6.50 | 0.30 | 0.33 | -2.24 | 2.74 | 3.40 | -2.69 | -2.10 | -4.56 | 0.1 | 4.00 |
|  | 0-2000m | -2.30 | -3.30 | 6.19 | 1.44 | 5.20 | -1.70 | -2.80 | -7.43 | 3.09 | 7.20 | 0 | 0.04 | -2.59 | 2.58 | 3.60 | -3.31 | -2.56 | -5.16 | -0.39 | 4.40 |

S4 Table: Table showing mean, median, standard deviation, 25^th^ and 75^th^ percentiles of along-shelf transport (in Sv) for each season through shelf transects off Seal Rocks (S1), Newcastle (S2) and Sydney (S3) on the Hawkesbury Shelf. Shelf transects represent the inner shelf (0-100m), middle shelf (100-200m), outer-shelf (200-2000m) and the entire shelf (0-2000 m).
