# Supplementary material for: Transport variability over the Hawkesbury Shelf (31.5–34.5°S) driven by the East Australian Current
Source: PLoS One. 2020 Nov 5;15(11):e0241622. doi: 10.1371/journal.pone.0241622 (PMC7644073; doi:10.1371/journal.pone.0241622)
Supplement: S5 Table — (DOCX) [file pone.0241622.s011.docx]

|  | Shelf-Section (Isobath) | Summer | | | | | Autumn | | | | | Winter | | | | | Spring | | | | |
| --- | --- | --- | --- | --- | --- | --- | --- | --- | --- | --- | --- | --- | --- | --- | --- | --- | --- | --- | --- | --- | --- |
|  |  | Mean | Median | 25 % | 75 % | SD | Mean | Median | 25 % | 75 % | SD | Mean | Median | 25 % | 75 % | SD | Mean | Median | 25 % | 75 % | SD |
| S1x | 100 | 0.17 | 0.17 | 0.05 | 0.28 | 0.2 | 0.14 | 0.1 | -0.04 | 0.28 | 0.24 | 0.11 | 0.11 | -0.05 | 0.25 | 0.18 | 0.11 | 0.09 | -0.01 | 0.22 | 0.16 |
|  | 200 | 1.3 | 1.49 | 0.35 | 1.05 | 0.8 | 0.68 | 0.79 | 0.09 | 0.89 | 1.04 | 0.81 | 0.82 | -0.06 | 0.76 | 0.74 | 0.65 | 0.57 | 0.02 | 0.78 | 0.91 |
|  | 2000 | 7.71 | 8.36 | 1.97 | 13.76 | 6.2 | 3.37 | 2.45 | -0.02 | 6.63 | 4.93 | 3.55 | 3.74 | 0.05 | 6.82 | 4.29 | 4.43 | 2.59 | -0.23 | 9.37 | 6.12 |
|  | 100-2000 | 9.19 | 10.02 | 0.51 | 14.52 | 6.4 | 4.19 | 3.34 | 0.16 | 7.29 | 5.43 | 4.47 | 4.67 | 0.69 | 7.16 | 4.43 | 5.19 | 3.25 | -0.27 | 10.30 | 6.78 |
|  |  |  |  |  |  |  |  |  |  |  |  |  |  |  |  |  |  |  |  |  |  |
| S2x | 100 | -0.02 | -0.02 | -0.05 | 0.02 | 0.06 | -0.02 | -0.02 | -0.06 | 0.02 | 0.07 | -0.03 | -0.03 | -0.06 | 0.01 | 0.05 | -0.04 | -0.04 | -0.06 | -0.01 | 0.04 |
|  | 200 | 0.13 | -0.09 | -0.47 | 0.38 | 0.60 | 0.30 | -0.05 | -0.63 | 0.46 | 0.58 | -0.24 | -0.1 | -0.43 | 0.17 | 0.62 | 0.07 | -0.18 | -0.49 | -0.02 | 0.64 |
|  | 2000 | 1.38 | 2.43 | 0.41 | 10.02 | 6.70 | 0.69 | 1.79 | -3.18 | 8.22 | 7.42 | 2.54 | 2.57 | 3.19 | 6.62 | 4.33 | 3.00 | 2.65 | 0.24 | 6.55 | 5.45 |
|  | 100-2000 | 1.30 | 2.32 | 0.23 | 10.53 | 7.27 | 0.70 | 1.72 | -3.44 | 10.33 | 7.80 | 2.41 | 2.44 | 3.13 | 6.44 | 4.56 | 2.75 | 2.36 | -0.46 | -6.56 | 5.56 |
|  |  |  |  |  |  |  |  |  |  |  |  |  |  |  |  |  |  |  |  |  |  |
| S3x | 100 | 0.01 | -0.01 | -0.04 | 0.04 | 0.06 | 0.00 | 0.01 | -0.05 | 0.05 | 0.08 | -0.05 | -0.04 | -0.01 | 0.01 | 0.08 | -0.01 | 0.01 | -0.03 | 0.02 | 0.05 |
|  | 200 | 0.19 | 0.16 | 0.35 | 1.05 | 0.49 | 0.27 | 0.46 | 0.09 | 0.89 | 0.69 | -0.14 | -0.14 | -0.06 | 0.76 | 0.59 | 0.28 | -0.24 | 0.02 | 0.78 | 0.39 |
|  | 2000 | 0.58 | 0.06 | -5.54 | 4.08 | 4.98 | 1.36 | 2.30 | -2.32 | 6.13 | 6.00 | -1.51 | -1.70 | -4.83 | 2.28 | 5.04 | 0.10 | -0.57 | -2.84 | 1.14 | 4.29 |
|  | 100-2000 | 0.76 | 0.21 | -3.46 | 4.23 | 5.22 | 1.62 | 2.77 | -2.08 | 6.45 | 6.51 | -1.67 | -1.88 | -5.14 | 2.37 | 5.38 | 0.34 | -0.80 | -2.76 | 1.59 | 4.56 |

S5 Table: Table showing mean, median, standard deviation, 25^th^ and 75^th^ percentiles of across-shore transport (in Sv) for each season through and each season through three along shelf transects off Seal Rocks (S1x), Newcastle (S2x) and Sydney (S3x) on the Hawkesbury Shelf across 3 isobaths, 100m, 200m and 2000m and the total 100-2000m.
